# Barcoded mutant library enables high-throughput functional genomics in a filamentous fungus

**DOI:** 10.1073/pnas.2616888123

**Published:** 2026-08-25

**Authors:** Lori B. Huberman, José M. Villalobos-Escobedo, Jeffrey M. Skerker, Ran Shi, Adriana M. Rico-Ramírez, Catharine A. Adams, Adam P. Arkin, Adam M. Deutschbauer, N. Louise Glass

**Affiliations:** ^a^Plant Pathology and Plant-Microbe Biology Section, School of Integrative Plant Science, Cornell University, Ithaca, NY 14853; ^b^ Department of Plant and Microbial Biology, University of California Berkeley, Berkeley, CA 94720; ^c^ Environmental Genomics and Systems Biology Division/Biosciences Area, Lawrence Berkeley National Laboratory, Berkeley, CA 94720; ^d^ Tecnologico de Monterrey, Institute for Obesity Research, Monterrey, NL 64700, México; ^e^ Department of Bioengineering, University of California Berkeley, Berkeley, CA 94720

**Keywords:** *Trichoderma*, barcoded mutant library, high-throughput functional genomics, high-throughput screen, filamentous fungi

## Abstract

Advances in sequencing technology enabling rapid and inexpensive whole-genome sequencing highlight how few genes are functionally characterized. This problem is particularly acute in filamentous fungi, where even in the best studied organisms upward of half of genes are poorly characterized or unannotated. High-throughput tools to identify gene function exist for single-celled organisms, like yeast and bacteria. However, filamentous fungi present challenges to high-throughput gene characterization, including low transformation efficiency and multinucleate cells. Filamentous fungi are critical components of nutrient cycling in ecosystems, form symbioses with plants that improve nutrient uptake, and are devastating human, plant, and animal pathogens causing millions of deaths and substantial crop loss each year. Thus, it is critical to overcome challenges to rapid gene characterization in filamentous fungi. We generated a library of hundreds of millions of uniquely barcoded plasmids containing a broad host-range drug resistance marker for ectopic insertion into filamentous fungal genomes by *Agrobacterium tumefaciens*. We then optimized *A. tumefaciens* mediated transformation of the biocontrol agent *Trichoderma atroviride* and made an insertional mutagenesis library containing 83,311 barcoded insertions, disrupting 5,331 of 11,863 predicted genes. This library enables high-throughput screens to rapidly connect genotype to phenotype. Quantifying relative barcode abundance in the pooled library before and after exposure to experimental conditions identified candidate genes and recovered known pathway components in amino acid biosynthetic, fructose utilization, and xylose utilization pathways. This resource establishes a scalable platform for high-throughput functional genomics in filamentous fungi, enabling investigations of fungal biology to improve medical outcomes, biotechnology, and sustainable agriculture.

## Significance

Filamentous fungi are critical ecosystem components that cycle nutrients and form plant symbioses. Some are also pathogens that cause millions of human deaths each year, threaten bat and amphibian species with extinction, and cause famines. Thus, it is important to understand the role of fungal genes. We developed tools to rapidly connect genotype to phenotype in filamentous fungi. We generated a library of tens of thousands of barcoded mutants in the biocontrol agent *Trichoderma atroviride*, which we used to identify genes involved in amino acid biosynthesis and sugar utilization. The tools we developed are applicable to a broad range of filamentous fungi and enable investigations to identify treatments for fungal infections of humans, animals, and plants and improve biotechnology applications.

Filamentous fungi have crucial roles in ecosystem dynamics, biotechnology, human disease, and agriculture. They degrade complex carbon sources, including lignocellulosic biomass and plastics and contribute to carbon cycling in the environment and bioremediation (1, 2). They also produce a vast array of secondary metabolites (3) and can act as protein and bioproduct factories (4), making them critical players in biotechnology. Some filamentous fungi are devastating pathogens that cause millions of human deaths each year (5), threaten bat and amphibian species with extinction (6, 7), and cause the loss of 30% of crops each year (8). Despite their ecological, biotechnological, medical, and agricultural relevance, most fungal genes remain uncharacterized and available methods to functionally characterize fungal genes are laborious.

High-throughput methods to connect genotype to phenotype exist in single celled organisms, like yeast and bacteria (9–11). One of the most effective methods of high-throughput functional genomics enabling systematic discovery of gene function is randomly barcoded transposon or transfer DNA (TDNA) insertional mutagenesis. In this method, transposons or TDNA from *Agrobacterium tumefaciens*-mediated transformation (ATMT) carrying unique DNA barcodes and a selectable marker are randomly inserted across the genome of a target organism. Sequencing the library allows each barcode to be associated with its insertion location. Exposing the pooled mutant library to conditions of interest enables parallel fitness assays of thousands of insertion mutants by quantitative sequencing to measure changes in the relative abundance of barcodes in the population across experimental conditions (9–11).

Although high-throughput functional genomic strategies have significantly accelerated gene annotation and systems-level understanding in unicellular microbes, they remain a significant challenge in filamentous fungi. The complexity of multinucleate mycelial growth in these organisms, combined with low transformation efficiencies and limited genetic tools, has hindered the development of high-throughput functional genomics approaches (12, 13). To generate tools for filamentous fungi, we adapted a high-efficiency randomly barcoded insertional mutagenesis sequencing pipeline (RB-TDNAseq) developed for a basidiomycete yeast (11) and applied it to *Trichoderma atroviride*, a biocontrol agent that promotes plant growth and suppresses phytopathogenic fungi (14). Like many other filamentous fungi, *Trichoderma* species lack tools for genome-wide functional characterization. Additionally, the ecophysiological diversity of *Trichoderma* species and functional plasticity of *Trichoderma* genes (14) can limit effectiveness of annotating genes by homology. We first constructed large-scale plasmid libraries with a selectable marker tagged with hundreds of millions of unique barcodes for ectopic integration into the *T. atroviride* genome via ATMT. Using an optimized ATMT protocol, we generated a library of 83,311 barcoded insertions, disrupting 5,331 of the 11,863 predicted genes in the *T. atroviride* genome.

As a proof-of-principle, we performed high-throughput screens using the *T. atroviride* barcoded mutant library to identify genes associated with amino acid biosynthetic, fructose utilization, and xylose utilization pathways. Combining the fitness scores of barcoded mutants in the *T. atroviride* library exposed to xylose with transcriptomic analysis provided robust evidence for roles of genes in xylose utilization. Our results demonstrate a scalable technique for genome-wide functional analysis in filamentous fungi applicable to a wide range of fungal species, opening new avenues for fundamental and applied research with medical, agricultural, and biotechnological applications in these multicellular, multinucleate organisms.

## Results

### Developing Tools to Construct Genome-Wide Insertional Mutagenesis Libraries.

RB-TDNAseq was developed to generate a barcoded insertional mutant library in the basidiomycete yeast *Rhodosporidium toruloides* (11). However, using RB-TDNAseq in filamentous fungi has several challenges relative to most unicellular microbes, including low transformation efficiencies, relatively large genomes (~40 to 150 Mb in a typical filamentous fungal genome compared to 20.7 Mb for *R. toruloides*, 12.1 Mb for *Saccharomyces cerevisiae*, or 0.1 to 10 Mb in bacteria), increased gene content, larger intergenic regions, and multinucleate cells. To develop libraries of uniquely barcoded filamentous fungal mutants for high-throughput screens, we first created a library of plasmids containing a drug resistance cassette, each tagged with a unique DNA barcode in a plasmid backbone optimized for ATMT (*SI Appendix*, Fig. S1) (15). We used a hygromycin resistance cassette suitable for transformation in a variety of filamentous fungi (16) as a selectable marker. We optimized an established Golden Gate Cloning protocol (17) for high efficiency cloning, enabling the generation of a library of an estimated 1 billion uniquely barcoded plasmids in *Escherichia coli* [according to a Chao estimation of true population size (9, 18)] (Dataset S1). We then optimized plasmid transformation of an *A. tumefaciens* strain (EHA 105), resulting in a library containing an estimated 290 million uniquely barcoded plasmids with a hygromycin resistance marker (*SI Appendix, SI Materials and Methods* and Dataset S1).

Generating insertional mutagenesis libraries in filamentous fungi with tens of thousands of insertions requires a high-efficiency transformation protocol that can be performed in a high-throughput manner. ATMT is possible in most fungal species (19) and is more amenable to high-throughput implementation than other fungal transformation protocols (i.e., protoplast transformation). We optimized ATMT of *T. atroviride* asexual spores (conidia) by altering the concentration and pH of the *A. tumefaciens* induction media buffer, the concentration of the metabolite (acetosyringone) that induces TDNA transfer by *A. tumefaciens*, the age of the *T. atroviride* conidia, the length of coincubation between *A. tumefaciens* and *T. atroviride*, and the temperature at which the coincubation was performed (see *SI Appendix, SI Materials and Methods*; https://www.protocols.io/view/transformation-protocol-atmt-trichoderma-atrovirid-261gekk87g47/v1). Adjusting these variables increased transformation efficiencies to ~600 transformants per transformation event.

**Fig. 1.**
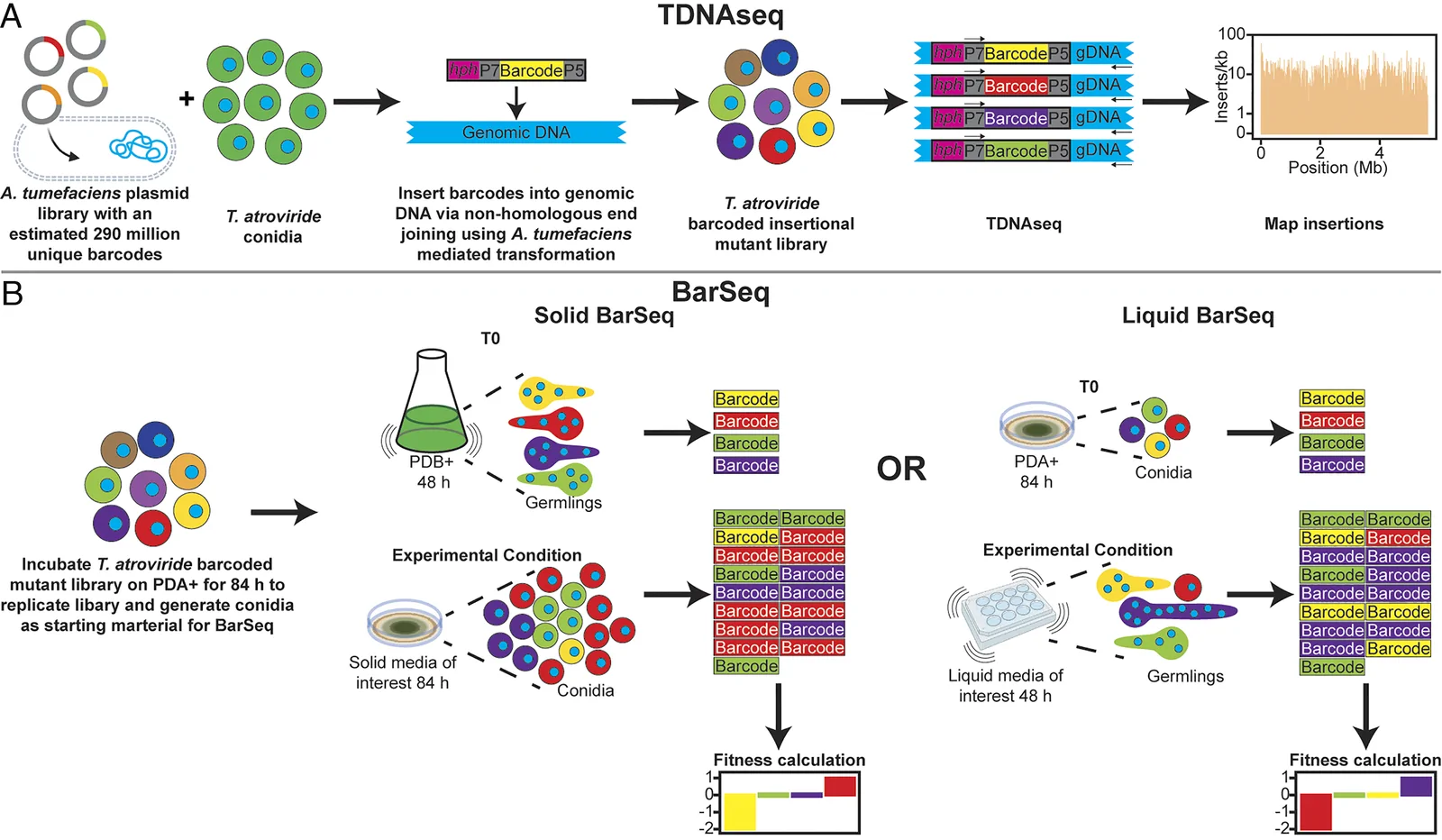
Schematic of RB-TDNAseq in *T. atroviride*. (*A*) Generation of a *T. atroviride* barcoded insertional mutant library using *A. tumefaciens*-mediated transformation. Plasmids containing drug resistance cassettes (*hph*), which confer hygromycin resistance, tagged with an estimated 1 billion unique 20 bp DNA barcodes, flanked by common PCR priming sites (P7 and P5), were generated using Golden Gate Cloning and introduced into *E. coli*. These plasmids were then extracted from *E. coli* and transformed into *A. tumefaciens*, creating a library of an estimated 290 million uniquely barcoded plasmids for *T. atroviride* transformation. Following selection of transformed *T. atroviride* homokaryotic transformants using hygromycin resistance, genomic DNA was extracted, and insertion sites were sequenced to map the barcodes to their respective genomic locations. (*B*) Conidia from the *T. atroviride* barcoded insertional mutant library were inoculated into both liquid and solid media containing different nutrient conditions and incubated at 28 °C. After the growth period, germinated conidia (liquid media) or newly matured conidia (solid media) were harvested, DNA was extracted, and the barcodes were amplified using PCR. These barcodes were then sequenced, and the relative abundance of the barcodes was quantified as a proxy for the relative abundance of each mutant in the population. Because each barcode was associated with a genomic position (described in *A*), fitness could be calculated for each disrupted gene. For more detailed BarSeq assay diagrams, see *SI Appendix*, Fig. S6. (Note: Cartoons are not drawn to scale).

Conidia of many *Trichoderma* species are uninucleate (20), enabling the recovery of homokaryotic transformants following ATMT. However, even with the addition of antibiotics, *A. tumefaciens* DNA was present in library preparations. To reduce contamination, we forced *T. atroviride* transformants to invasively grow through selective top agar prior to collecting homokaryotic conidia from transformants (*SI Appendix*, Fig. S2). *A. tumefaciens* cells were trapped under the top agar, resulting in relatively clean separation of *T. atroviride* transformants from *A. tumefaciens* cells. We collected transformed conidia and made frozen stocks of library aliquots to reduce the need for library propagation, which we expect would reduce library diversity. A schematic of the transformation protocol is shown in Fig. 1*A*.

### Insertions Are Broadly Distributed Across the *T. atroviride* Genome.

**Fig. 2.**
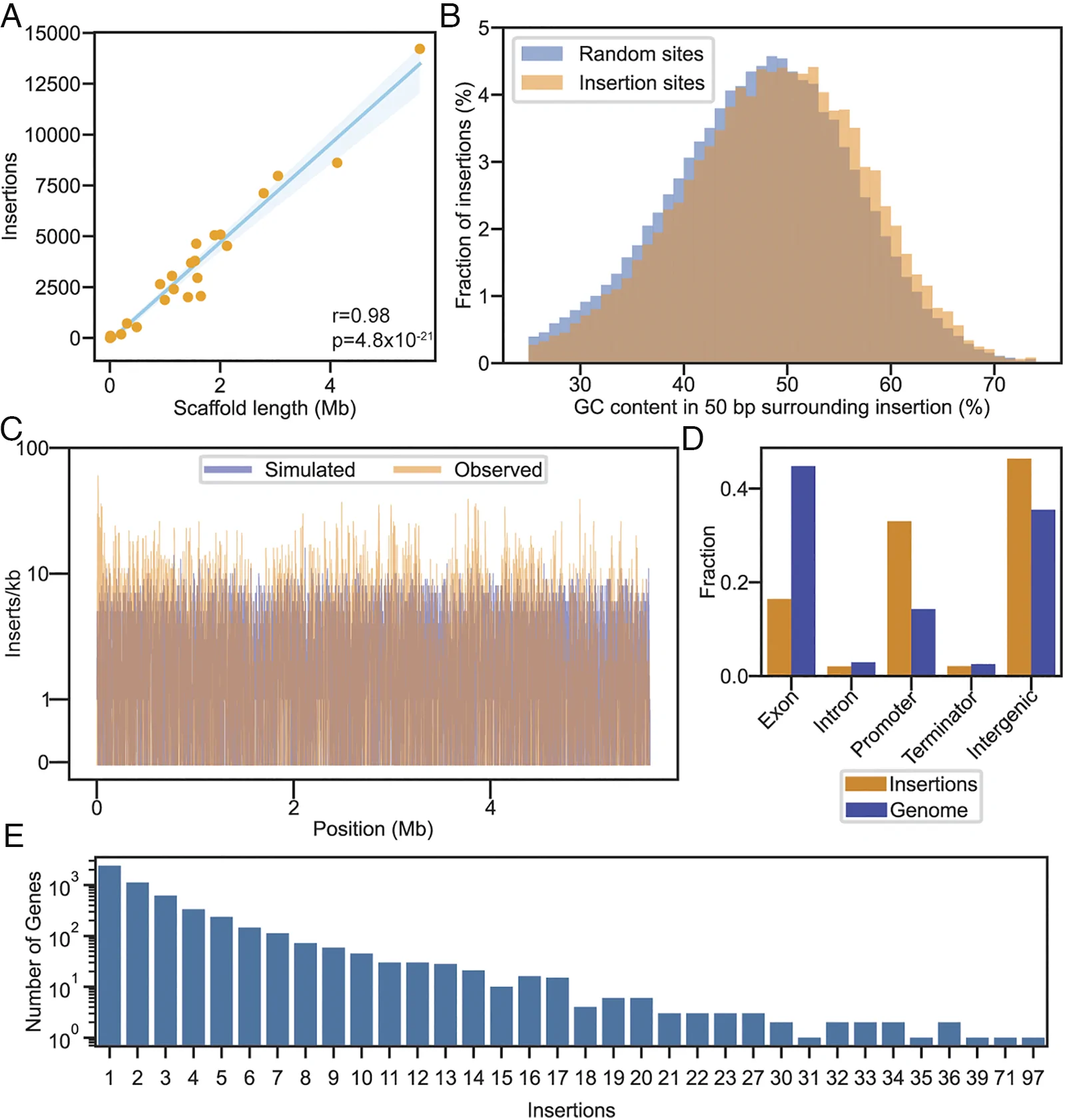
The 83,311 uniquely barcoded TDNA insertions are broadly distributed across the *T. atroviride* genome. (*A*) Number of insertions in each scaffold of the *T. atroviride* genome relative to the length of each scaffold. The *T. atroviride* genome has 29 scaffolds ranging in size from 4.7 kb to 1.5 Mb (22). Line is a linear regression fit, and band is 95% CI, with r = 0.98 and *P* = 4.8 × 10^−21^. (*B*) Percent of insertions in regions of the genome with a particular GC content (orange) compared to the percent of the genome with a particular GC content (blue). The GC content of an insertion site is defined as the 50 bp region surrounding the insertion site. There is a slight insertion bias in regions of higher GC content (Welch’s *t* test, *P* = 6 × 10^−219^). (*C*) Number of insertions per kb in 500 bp windows across the largest scaffold (ABDG02000027; 5.6 Mb) (orange) compared to a simulation of random insertion density across the largest scaffold (blue). (*D*) Fraction of insertions found in a genomic feature (orange) compared to the fraction of the genome annotated with a particular genomic feature (blue). The fractions of insertions in genomic features are significantly different from the fractions of the genome in genomic features (χ^2^ statistic = 38,537; *P* = 0). (*E*) Number of predicted genes that contain from one to 97 insertions.

Following ATMT, hygromycin-resistant *T. atroviride* transformants were inoculated onto potato dextrose agar (PDA) plates supplemented with amino acids, nucleic acids, and vitamins (PDA+) and allowed to sporulate. Conidia were collected, and a modified version of the KAPA DNA sequencing library preparation protocol for Illumina sequencing was used in which the PCR amplification step was optimized to highly enrich for DNA fragments containing a barcoded insertion (21) (*SI Appendix, SI Materials and Methods*). After adapter ligation to fragmented DNA, we performed two PCR enrichment steps for genomic DNA fragments containing a barcode with a forward primer immediately upstream of the barcode within the TDNA insertion cassette and a reverse primer in the Illumina adapter that was ligated to the *T. atroviride* genomic DNA downstream of the insertion site (*SI Appendix*, Fig. S1). The randomly barcoded TDNA (RB-TDNA) libraries were sequenced on the Illumina platform allowing the association of the barcodes with their insertion locations across the *T. atroviride* genome (Figs. 1*A* and 2 and Dataset S2).

The RB-TDNA insertion site sequencing data showed 54% of reads mapped to barcoded insertion sites in the *T. atroviride* genome (*SI Appendix*, Fig. S3*A*). Thirteen percent of reads mapped to the ATMT plasmid, likely due to a combination of incorrect processing of the plasmid during ATMT, concatemer formation due to insertion of multiple TDNAs in a single location in the genome, or any remaining contamination of the library with *A. tumefaciens* cells containing unprocessed ATMT plasmids. The remaining 33% of reads did not contain a barcode, likely due to mispriming of PCR amplification primers either to the *T. atroviride* or *A. tumefaciens* genomes (*SI Appendix*, Fig. S3*A*).

We detected 149,238 barcodes in our sequenced population. Of these, 5,086 barcodes were identified at multiple locations in the genome, and the genomic location of 414 barcodes could not be definitively determined. Additionally, 60,427 barcodes mapped exclusively to the ATMT plasmid. This left 83,311 barcodes that mapped to a unique *T. atroviride* genomic location. Of these, 78,771 mapped exclusively to the genome. The remaining 4,540 mapped to both a single genomic location and the insertion sequence due to concatemer formation caused by insertions in the same direction (4,494 barcodes) or opposite direction in the genome (46 barcodes) (*SI Appendix*, Fig. S3*B* and Dataset S2).

The 83,311 barcoded insertions in the *T. atroviride* genome were evenly distributed among each of the 29 scaffolds (R = 0.98; *P* = 4.8 × 10^−21^) (22) with a slight bias toward regions with higher GC content (Welch’s *t* test, *P* = 6 × 10^−219^) (Fig. 2 *A* and *B* and Dataset S3). The insertion bias toward regions of higher GC content could be due to AT-rich heterochromatin (23) that may be less accessible to insertion or because AT-rich regions are more repetitive, making mapping insertions to these regions more challenging. To examine the distribution of insertion sites in greater detail, we plotted the insertion profiles for all scaffolds greater than 500 kb in length and compared these insertion profiles to a simulation of random insertions across the genome. Although insertions were broadly distributed across these 17 scaffolds, the insertion distribution was not random, leading to hot and cold spots (Wald-Wolfowitz runs test on 500 bp windows across the 17 largest scaffolds, *P* < 1.5 × 10^−16^) (Fig. 2*C* and *SI Appendix*, Fig. S4). Insertion hot and cold spots may indicate unidentified genomic features that modulated accessibility of TDNA insertion into chromosomal DNA.

Biases could also be caused by insertions into genomic features that modulate transformant fitness. To assess this possibility, we evaluated differences in the relative fraction of insertion locations in the library across five genomic features (exon, intron, promoter, terminator, and intergenic regions) compared to the relative fraction of those genomic features in the genome. The library had more insertions in predicted promoter sequences and intergenic regions and fewer insertions in exons, introns, and terminators than would be expected by chance (χ^2^ statistic = 38,537; *P* = 0) (Fig. 2*D* and Dataset S3). The bias away from insertions in coding regions is likely due, in part, to selection against insertions in essential genes, genes that cause strongly deleterious phenotypes when mutated, or genes required for invasive growth of transformants through the top agar. Additionally, because promoter regions and transcriptional start sites tend to have lower nucleosome occupancy, while exons tend to have higher nucleosome occupancy, this genomic feature bias may point to differences in accessibility to TDNA insertions (24, 25).

Despite a bias away from insertions in exons and introns, the library still contained 15,412 barcoded insertions in open reading frames, corresponding to the disruption of 5,331 of the 11,863 predicted genes in the *T. atroviride* genome (Dataset S2) (22). Of these, 2,401 genes had a single insertion, 1,116 genes had two insertions, 1,814 genes had at least three insertions, and 238 genes had at least 10 insertions (Fig. 2*E* and Dataset S3). The number of insertions per gene did not correlate with gene length (*SI Appendix*, Fig. S5). When mapping insertions, we assumed barcodes mapped to the same location whose sequences were within a Levenshtein edit distance (26) of five were the same barcode. However, several genes had many barcodes with substantially different sequences that mapped to a single location, including a gene encoding a predicted histidine kinase response regulator (TRIATDRAFT_149807) and a gene predicted to encode a Ras GTPase activating protein (TRIATDRAFT_137944) (Dataset S3). Of the 97 insertions in TRIATDRAFT_149807, 93 occurred in a single genomic location, while of the 71 insertions in TRIATDRAFT_137944, 66 were in a single genomic location (Dataset S2). While it is possible these genomic locations are particularly prone to double strand breaks and these sites truly have many insertions, it is more likely PCR amplification errors during the TDNAseq library prep or sequencing errors during Illumina sequencing altered barcode sequences beyond a Levenshtein edit distance of five, artificially inflating insertion numbers in these genes.

Genes with multiple barcoded insertions likely correspond to multiple individual mutants rather than a single mutant with multiple disruptions. However, because TDNAseq only sequences approximately 100 bp of genomic DNA following the insertion, we cannot state this with certainty. Although it is possible transcription of a gene fragment remaining upstream or downstream of the TDNA insert within a coding region could result in a hypomorph, hypermorph, or neomorph allele, most disruptions likely result in loss-of-function mutations. Regardless, since pooled, high-throughput screens generate fitness data on all insertional mutants, fitness phenotypes could be further investigated to determine whether a clean deletion or gene truncation phenocopies the insertional mutant.

### Fitness Assays Performed on the Barcoded Mutant Library Identified Predicted Amino Acid Biosynthesis Genes.

The goal of constructing the barcoded insertional mutant library was to identify functions for uncharacterized genes. Barcoded mutant libraries have been highly effective at identifying genes in amino acid biosynthetic pathways in yeast and bacteria (11, 27). We therefore tested the ability of the *T. atroviride* barcoded mutant library to identify auxotrophic mutants via high-throughput fitness assays. We exposed the library either to minimal media, complete media, or complete media lacking cysteine and measured the change in the relative abundance of unique barcodes (representing mutants containing insertions in the library) between the starting population and the population after exposure to the experimental condition of interest (BarSeq) (Fig. 1*B* and *SI Appendix*, Fig. S6). For genes with a single insertion, gene fitness was computed as the log_2_(fold change) in relative abundance of the barcode associated with the gene insertion in the test condition compared to the relative barcode abundance in the starting population. For genes with multiple insertions in the library, we calculated gene fitness by averaging the log_2_(fold change) in relative abundance of all barcodes associated with insertions in that gene in the test condition compared to the starting population (9–11).

To measure gene fitness, we inoculated frozen aliquots of the library on PDA+ plates, incubated them for 84 h, and harvested and pooled the resulting conidia. We then split the conidial pool into an aliquot that was used to measure library diversity at the start of the experiment (T0) and experimental aliquots. The T0 aliquot was inoculated into liquid potato dextrose broth (PDB) supplemented with amino acids, nucleic acids, and vitamins (PDB+) and grown for 48 h. The mycelia were then harvested to measure the library diversity (Fig. 1*B* and *SI Appendix*, Fig. S6). The experimental conidial aliquots were inoculated onto agar plates containing either Vogel’s minimal medium (VMM) (28) with 1% glucose (minimal medium); VMM supplemented with all 20 amino acids, adenine, uracil, p-aminobenzoic acid, and inositol with 1% glucose (complete medium); or VMM supplemented with adenine, uracil, p-aminobenzoic acid, inositol, and all amino acids except cysteine with 1% glucose (complete medium without cysteine). These plates were incubated for 84 h until mature conidia were produced. We then harvested and performed BarSeq on these mature conidia.

We performed our fitness analysis using fitness calculations and statistical methods developed for analyzing bacterial BarSeq data (9) and updated for fungal barcoded libraries (11, 29). To reduce false negatives caused by an insertion in the very beginning or end of a gene, we computed fitness scores using insertions in the middle 90% of coding regions. To reduce false positives, we required that barcodes have at least three counts across the T0 and experimental condition to be included in our analysis. To compute gene fitness scores, we required at least 20 counts across all barcoded insertions in a gene. Barcodes that did not meet all these criteria were not included in our fitness analysis. We used multiple methods to calculate statistical significance of fitness scores (9, 11, 29). The first was a *t*-like test statistic that estimates the reliability of fitness scores (9). The second was a *P*-value calculated by treating the *t*-like test statistic as a true *t*-statistic after Benjamini–Hochberg multiple hypothesis correction (11, 29). This *P*-value is a false discovery rate.

These criteria resulted in fitness scores for 2,858 genes in minimal medium, 2,843 genes in complete medium, and 2,829 genes in complete medium without cysteine (Dataset S4). To assess the correlation between BarSeq replicates, we compared the gene fitness scores of each replicate within the complete medium, complete medium without cysteine, and minimal medium experiments. The R-values of replicate comparisons ranged from 0.52 to 0.57 for complete medium, 0.6 to 0.64 for complete medium without cysteine, and 0.59 to 0.75 for minimal medium (*SI Appendix*, Fig. S7). These correlations suggest that while the replicates were not identical, they were broadly similar.

**Fig. 3.**
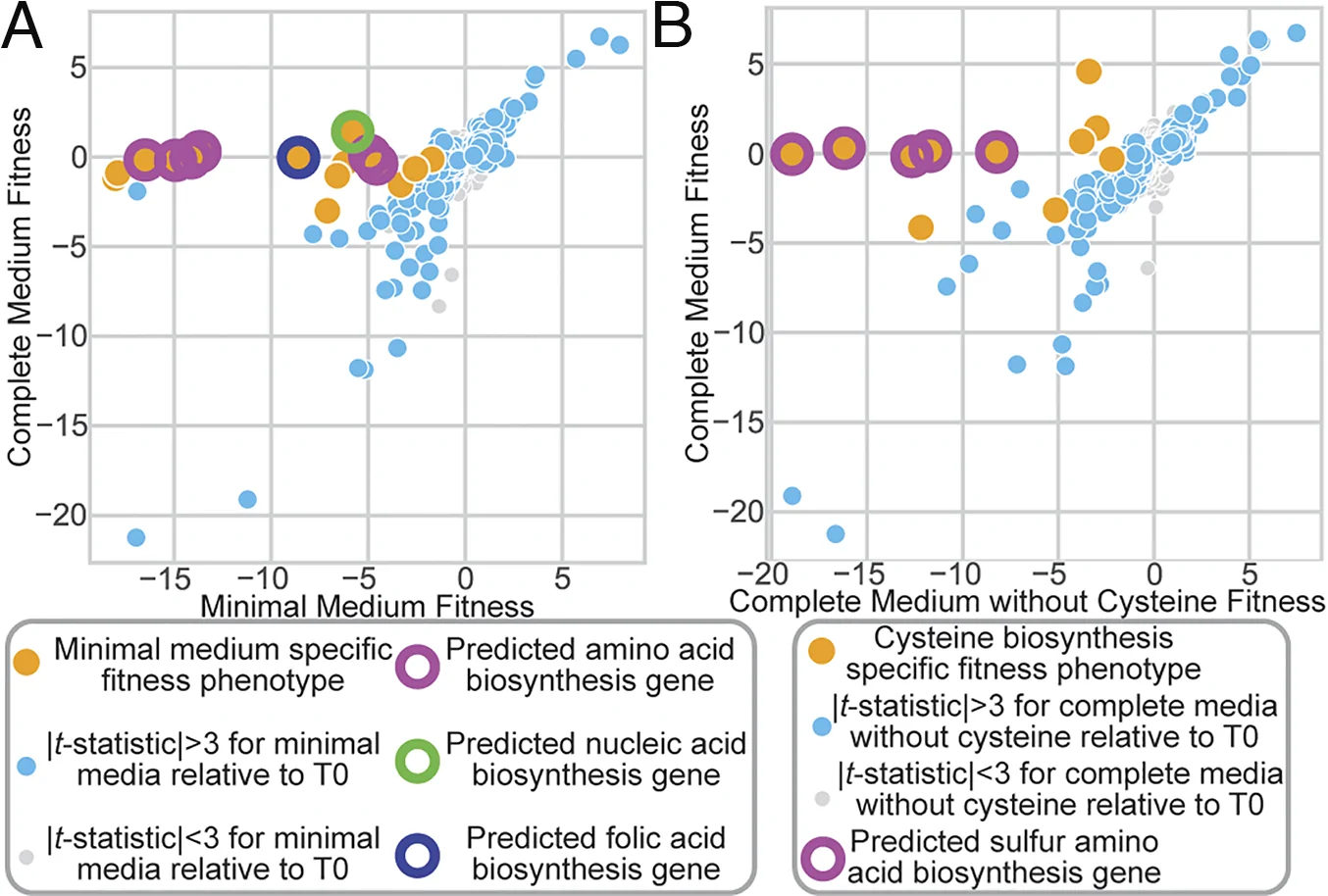
Identification of amino acid biosynthesis genes using BarSeq. (*A*) Fitness scores relative to T0 of 2,797 genes obtained by growing the insertional mutant library on solid minimal medium and complete medium for 84 h; conidia were subsequently harvested for BarSeq. Genes with a minimal medium-specific fitness phenotype (i.e., minimal medium relative to T0 and minimal medium relative to complete medium fitness scores below −1.5 with a *t*-statistic < −3) are indicated in orange. All other genes with minimal medium relative to T0 fitness scores with |*t*-statistic| > 3 are indicated in light blue. Genes with a minimal medium-specific fitness phenotype predicted to play a role in amino acid biosynthesis are outlined with magenta circles. Genes with a minimal medium-specific fitness phenotype predicted to play a role in nucleic acid biosynthesis are outlined with green circles. Genes with a minimal medium-specific fitness phenotype predicted to play a role in folic acid biosynthesis are outlined with dark blue circles. Genes with minimal medium relative to T0 fitness scores that are not statistically significant (|*t*-statistic| < 3) are indicated in gray. (*B*) Fitness scores relative to T0 of 2,772 genes obtained by growing the insertional mutant library on solid complete medium or complete medium without cysteine for 84 h; conidia were subsequently harvested for BarSeq. Genes with a complete medium without cysteine-specific fitness phenotype (i.e., complete medium without cysteine relative to T0 and relative to complete medium fitness scores below −1.5 with *t*-statistic < −3) are indicated in orange. All other genes with complete medium without cysteine relative to T0 fitness scores with |*t*-statistic| > 3 are indicated in blue. Genes with a complete medium without cysteine-specific fitness phenotype predicted to play a role in sulfur amino acid biosynthesis are outlined with magenta circles. Genes with complete medium without cysteine relative to T0 fitness scores that are not statistically significant (|*t*-statistic| < 3) are indicated in gray.

We first looked for mutants in nucleic acid, vitamin, and all amino acid biosynthesis pathways by comparing the gene fitness in complete medium to gene fitness in minimal medium using two separately computed fitness scores. One was a fitness score computed by comparing barcode abundance in the experimental condition to the starting population (T0). The second fitness score was computed by comparing the barcode abundance in the experimental condition of interest to a control condition (i.e., complete media). We defined fitness scores as significant if they had a *t-*statistic greater than 3 or less than −3, as previously described (11). We defined genes with minimal medium-specific significant fitness defects as those with a fitness less than −1.5 in both the comparison between minimal medium and the starting condition to identify genes that cause growth defects on minimal medium and between minimal medium and complete medium to eliminate pleiotropic mutants. Seventeen genes (orange dots in Fig. 3*A*) met these criteria (Fig. 3*A*, *SI Appendix*, Fig. S8*A*, and Dataset S4).

We hypothesized these 17 genes would include genes with predicted roles in nucleic acid, vitamin, or amino acid biosynthesis. Insertions in TRIATDRAFT_139677, predicted to encode dihydropyrimidinase, which plays a role in pyrimidine biosynthesis, resulted in a fitness relative to T0 of −5.8 on minimal medium and 1.4 on complete medium (Fig. 3*A*, *SI Appendix*, Fig. S8*A*, and Dataset S4). TRIATDRAFT_77696 is predicted to encode para-aminobenzoate synthase, which may play a role in folic acid synthesis. Insertions in TRIATDRAFT_77696 resulted in a fitness relative to T0 of −8.6 on minimal medium and −0.029 on complete medium (Fig. 3*A*, *SI Appendix*, Fig. S8*A*, and Dataset S4). Insertions in six genes with predicted roles in amino acid biosynthesis caused significantly reduced fitness on minimal medium. Four of these genes have predicted roles in sulfur amino acid metabolism: TRIATDRAFT_297824 (siroheme synthase), TRIATDRAFT_45036 (homoserine O-acetyltransferase), TRIATDRAFT_223153 (sulfite reductase flavoprotein component), and TRIATDRAFT_137525 (cystathionine γ-synthase). The remaining two genes had predicted roles in arginine biosynthesis: TRIATDRAFT_302945 (Arg6 mitochondrial precursor) and TRIATDRAFT_44131 (acetylglutamate synthase) (Fig. 3*A*, *SI Appendix*, Fig. S8*A*, and Dataset S4).

Insertions in TRIATDRAFT_300639 resulted in the most severe fitness defect on minimal medium relative to T0 (−18) (Fig. 3*A*, *SI Appendix*, Fig. S8*A*, and Dataset S4). TRIATDRAFT_300639 is predicted to encode indoleamine 2,3-dioxygenase, which plays a role in tryptophan catabolism, but our fitness data suggest that TRIATDRAFT_300639 may play a role in amino acid biosynthesis. The remaining eight genes with minimal medium-specific fitness defects did not have a clear connection to amino acid metabolism. These genes encode a predicted phosphatidylinositol transfer protein (TRIATDRAFT_78942), three predicted transcription factors or proteins with DNA binding domains (TRIATDRAFT_288132, TRIATDRAFT_281078, and TRIATDRAFT_92406), two proteins with WD40 repeats (TRIATDRAFT_181383 and TRIATDRAFT_317751), and two hypothetical proteins (TRIATDRAFT_43604 and TRIATDRAFT_299984) (Fig. 3*A*, *SI Appendix*, Fig. S8*A*, and Dataset S4).

To test the specificity of our fitness assays, we asked whether we could identify genes in a specific amino acid biosynthesis pathway by comparing fitness on complete medium without cysteine to fitness on complete medium. We used a similar threshold for cysteine auxotrophs as we used for general amino acid, nucleic acid, and vitamin auxotrophs: fitness scores below −1.5 in complete medium without cysteine relative to T0 and relative to complete medium with *t*-statistics below −3. Eleven genes met these criteria, and five of the six genes with the most severe fitness defects on complete medium without cysteine relative to T0 had predicted roles in sulfur amino acid biosynthesis (Fig. 3*B*, *SI Appendix*, Figs. S8*B* and S9, and Dataset S4). These genes encoded for homoserine O-acetyltransferase (TRIATDRAFT_45036; fitness relative to T0 of −18.9 on complete medium without cysteine), sulfite reductase flavoprotein component (TRIATDRAFT_223153; fitness relative to T0 of −16.2 on complete medium without cysteine), siroheme synthase (TRIATDRAFT_297824; fitness relative to T0 of −12.7 on complete medium without cysteine), cysteine synthase (TRIATDRAFT_147947; fitness relative to T0 of −11.7 on complete medium without cysteine), and cystathionine γ-synthase (TRIATDRAFT_137525; fitness relative to T0 of −8.2 on complete medium without cysteine) (Fig. 3*B*, *SI Appendix*, Figs. S8*B* and S9, and Dataset S4). The remaining genes with significant fitness phenotypes on complete medium without cysteine had unclear roles in sulfur amino acid biosynthesis and included genes that are predicted to encode a 3,4-dihydroxy-2-butanone 4-phosphate synthase (TRIATDRAFT_150700), a NADH-ubiquinone oxidoreductase (TRIATDRAFT_148757), a DEAD box helicase (TRIATDRAFT_294184), a transcription factor (TRIATDRAFT_84050), dihydroorotase (TRIATDRAFT_139677), and a SWI-SNF chromatin-remodeling complex protein (TRIATDRAFT_171735) (Fig. 3*B*, *SI Appendix*, Fig. S8*B*, and Dataset S4).

### Fitness Assays Identified Genes Necessary to Utilize Fructose and Xylose.

Carbon source utilization is critical for fungal growth, development, and pathogenesis (1, 30). In *T. atroviride*, carbon sensing and utilization plays an important role in biocontrol (31). Thus, we used the barcoded insertional mutant library to identify genes associated with fructose and xylose utilization by comparing gene fitness on media containing fructose or xylose as the sole carbon source to gene fitness on media containing glucose as the sole carbon source. Because filamentous fungal development can proceed differently in solid versus liquid media, we performed fitness assays on the barcoded insertional mutant library in both solid and liquid media. We conducted solid media fitness assays to identify genes associated with fructose and xylose utilization in a similar fashion to the high-throughput screens for amino acid auxotrophs, except the experimental conidial aliquots were inoculated onto solid VMM plates containing 1% glucose, 1% fructose, or 1% xylose as the carbon source (Fig. 1*B* and *SI Appendix*, Fig. S6).

To perform fitness assays in liquid media, we inoculated frozen aliquots of the library on PDA+ plates and incubated them for 84 h until mature conidia formed. We then harvested and pooled the conidia. These conidia were split into an aliquot that was directly used to measure library diversity at the start of the experiment (T0) and paired experimental aliquots used to inoculate liquid VMM with either 1% glucose, 1% fructose, or 1% xylose as the carbon source. We grew the experimental aliquots in VMM 1% glucose, VMM 1% fructose, or VMM 1% xylose for 48 h, and BarSeq was performed on the resulting mycelia (Fig. 1*B* and *SI Appendix*, Fig. S6).

Like for the high-throughput screens for auxotrophic mutants, we required that barcodes have at least three counts across the T0 and experimental condition to be included in analysis. To compute a gene fitness in xylose, we required there be at least 20 counts across all barcodes representing insertions in the middle 90% of the gene. These criteria resulted in fitness scores for 3,137 genes in xylose in liquid media, 2,848 genes in xylose in solid media, 3,211 genes in glucose in liquid media, and 2,879 genes in glucose in solid media (Dataset S5). Counts representing insertions in relevant genes were low in our fructose dataset, so to compute a gene fitness on fructose, we only required at least 10 counts across all barcodes. These criteria resulted in fitness scores for 3,360 genes in fructose in liquid media, 3,165 genes in fructose in solid media, 3,418 genes in glucose in liquid media, and 3,193 genes in glucose in solid media (Dataset S5).

We first compared the reproducibility of solid media versus liquid media fitness assays by comparing fitness scores relative to T0 across replicates. The solid media carbon source experiments had similar reproducibility to the experiments comparing minimal and complete media. The R-values of replicate comparisons when we required 20 counts per gene ranged from 0.59 to 0.76 for glucose and 0.6 to 0.66 for xylose (*SI Appendix*, Fig. S10). As expected, the R-values of replicate comparisons when we only required 10 counts per gene were slightly lower: 0.44 to 0.72 for glucose and 0.51 to 0.56 for fructose (*SI Appendix*, Fig. S11). Like for the auxotrophy screens, the replicates in these carbon source screens were not very highly correlated but had clear similarity. The reproducibility of replicates in liquid media was much more limited. When we required 20 counts per gene, R-values of replicate comparisons were 0.06 to 0.12 for glucose and 0.02 to 0.1 for xylose (*SI Appendix*, Fig. S12). We saw a similar trend when we required only 10 counts per gene: R-values of replicate comparisons were 0.04 to 0.14 for glucose and 0.04 to 0.11 for fructose (*SI Appendix*, Fig. S13). The lack of similarity between replicates indicates that while it was possible to calculate fitness values from assays performed in liquid media, the liquid media BarSeq experiments were not very consistent.

**Fig. 4.**
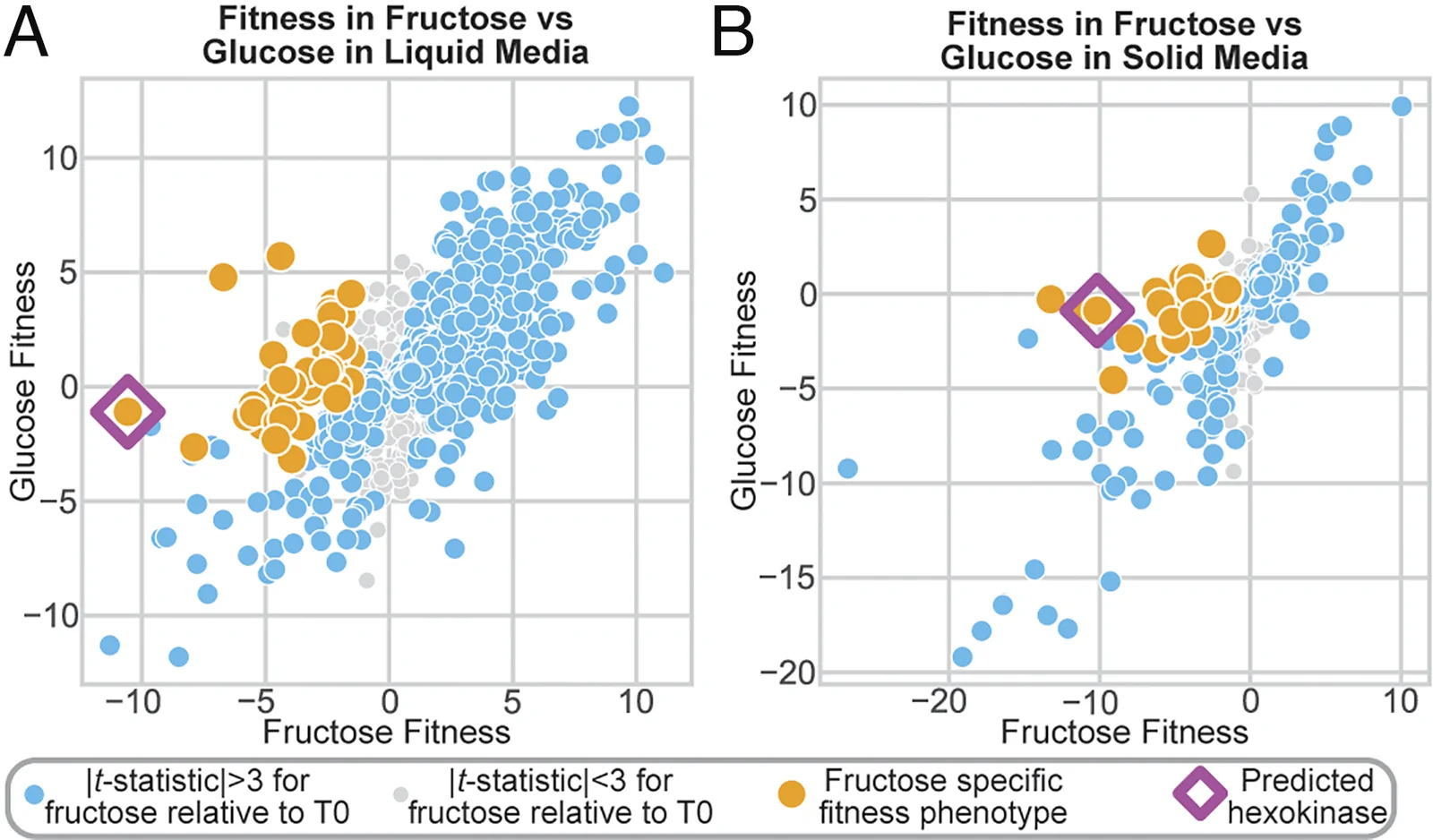
Identification of fructose utilization genes using BarSeq. (*A*) Fitness scores relative to T0 of 3,321 genes obtained by growing the insertional mutant library in liquid VMM with glucose or fructose as the sole carbon source for 48 h; germlings were subsequently harvested for BarSeq. Genes with a fructose-specific fitness phenotype (i.e., fructose relative to T0 and fructose relative to glucose fitness scores below −1.5 with a *t*-statistic < −3) are indicated in orange. A gene predicted to encode a hexokinase required for fructose utilization is outlined with a magenta diamond. All other genes with fructose relative to T0 fitness scores with |*t*-statistic| > 3) are indicated in blue. Genes with fructose relative to T0 fitness scores that are not statistically significant (|*t*-statistic| < 3) are indicated in gray. (*B*) Fitness scores relative to T0 of 3,117 genes obtained by growing the insertional mutant library on solid VMM media containing glucose or fructose as the sole carbon source and incubated for 84 h; conidia were subsequently harvested for BarSeq. Colors of data points on the graph are the same as in (*A*).

To identify genes with potential roles in fructose utilization, we compared the relative fitness of barcoded mutants on fructose to glucose. We defined genes with fructose-specific fitness phenotypes as those with fructose relative to T0 and fructose relative to glucose fitness scores less than −1.5 with *t*-statistics less than −3. Forty and 32 genes met these criteria in liquid and solid media, respectively (Fig. 4, *SI Appendix*, Fig. S14, and Dataset S5).

In other fungi, a gene encoding a hexokinase is known to be required for fructose catabolism (32). The gene predicted to encode this hexokinase (TRIATDRAFT_298279) had a fructose-specific fitness defect in both solid and liquid (fitness relative to T0 of −10.6 in liquid fructose media and −10.2 in solid fructose media) (Fig. 4, *SI Appendix*, Fig. S14, and Dataset S5). These data indicate high-throughput screens utilizing the barcoded mutant library can identify genes associated with carbon utilization.

Roles for other genes with significantly reduced fitness on fructose did not have known roles in fructose utilization or a clear connection to sugar metabolism more generally. Genes with the most negative fitness in liquid included genes predicted to encode an N-acetyl-γ-glutamyl-phosphate reductase (TRIATDRAFT_ 255445), an IMP-specific 5’-nucleotidase (TRIATDRAFT_137170), the CSN2 subunit of the COP9 signalsome (TRIATDRAFT_141643), a 3-oxoacyl-[acyl-carrier protein] reductase (TRIATDRAFT_217448), an adenine phosphoribosyl transferase (TRIATDRAFT_288146), and a dipeptidyl aminopeptidase (TRIATDRAFT_141550) (Fig. 4*A*, *SI Appendix*, Fig. S14, and Dataset S5). On solid media, genes with the most negative fitness included genes predicted to encode the Rho GTPase effector *BNI1* (TRIATDRAFT_168459), a dimethylaniline monooxygenase (TRIATDRAFT_158542), a transcription factor (TRIATDRAFT_191111), a methylenetetrahydrofolate reductase (TRIATDRAFT_33767), and a nucleobase transmembrane transporter (TRIATDRAFT_229348) (Fig. 4*B*, *SI Appendix*, Fig. S14, and Dataset S5).

**Fig. 5.**
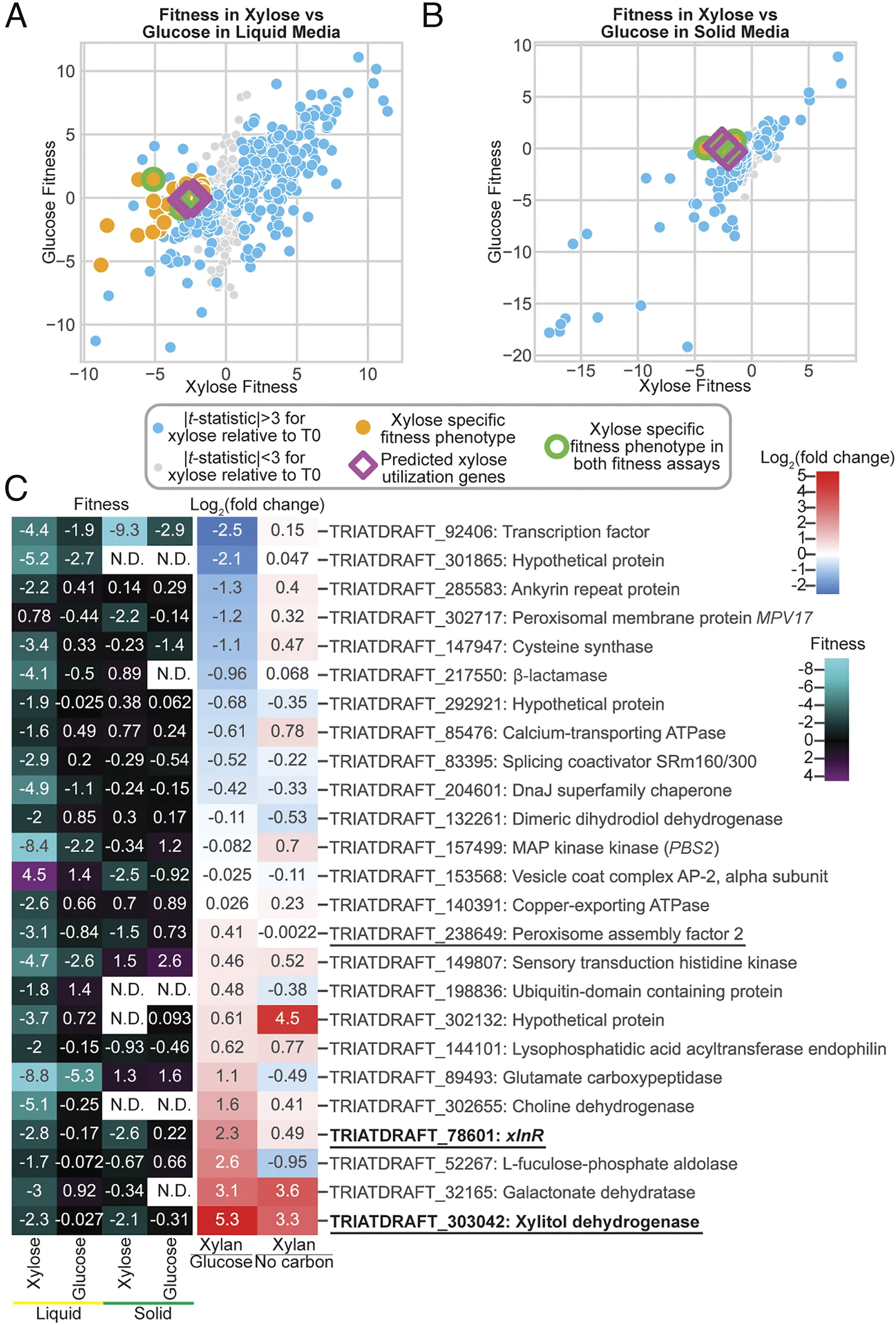
Identification of xylose utilization genes using BarSeq and comparison of fitness scores with expression data. (*A*) Fitness scores relative to T0 for 3,106 genes obtained by growing the insertional mutant library in liquid VMM with either glucose or xylose as the sole carbon source for 48 h; germlings were subsequently harvested for BarSeq. (*B*) Fitness scores relative to T0 for 2,805 genes obtained by growing the insertional mutant library on solid VMM media containing glucose or xylose as the sole carbon source and incubated for 84 h; conidia were subsequently harvested for BarSeq. Genes with xylose relative to T0 and xylose relative to glucose fitness scores below −1.5 with *t*-statistics < −3 are indicated in orange. Genes that met these criteria in both the (*A*) liquid and (*B*) solid media fitness assays are outlined with green circles. Genes predicted to play a role in xylose utilization are outlined with magenta diamonds. All other genes with xylose relative to T0 fitness scores with |*t*-statistic| > 3 are indicated in blue. Genes with xylose relative to T0 fitness scores that are not statistically significant (|*t*-statistic| < 3) are indicated in gray. (*C*) Heatmap of fitness relative to T0 (*Left*) or the log_2_(fold change) in expression (*Right*) of genes in *T. atroviride* with xylose relative to T0 and xylose relative to glucose fitness scores below −1.5 with *t*-statistics < −3 in either the (*A*) liquid or (*B*) solid fitness assay and that have average transcripts per million (TPM) of at least five in at least one condition. Fitness relative to T0 and log_2_(fold change) values are indicated on the heatmaps. RNAseq was performed during exposure to xylan, glucose, and carbon starvation (no carbon). BarSeq was performed on xylose and glucose in both liquid and solid media. Genes predicted to play a role in xylose utilization are indicated in bold. Genes that had xylose-specific fitness phenotypes in both solid and liquid media are underlined. N.D. = No data, indicating that no barcodes associated with that gene had at least three counts between the reference condition and test condition or that the total number of barcode counts across all insertions in the gene was below 20 counts in the reference and test condition.

We used similar criteria to identify genes necessary for xylose utilization. We defined genes with xylose-specific phenotypes as genes with xylose relative to T0 and xylose relative to glucose fitness scores less than −1.5 with *t*-statistics less than −3. These criteria identified 34 and eight genes with xylose-specific fitness phenotypes in liquid and solid media, respectively (Figs. 5 *A* and *B* and Dataset S5). Five genes had xylose-specific fitness phenotypes in both the liquid and solid media BarSeq experiments (Dataset S5). Two of these genes had predicted roles in xylose utilization (Fig. 5). TRIATDRAFT_78601 is homologous to XlnR, a transcriptional regulator responsible for activating genes necessary for xylose utilization in other ascomycete filamentous fungi (33–35), and showed a clear fitness defect in xylose relative to T0 (−2.8 in liquid media and −2.6 in solid media) (Fig. 5 and Dataset S5). A gene encoding a predicted xylitol dehydrogenase (TRIATDRAFT_303042) that is homologous to genes involved in xylose utilization (36) also had xylose-specific fitness defects (fitness relative to T0 of −2.3 in liquid media and −2.1 in solid media) (Fig. 5 and Dataset S5). Predicted roles for the other three genes with xylose-specific fitness defects in both liquid and solid media in xylose utilization were less clear: TRIATDRAFT_38402 (fitness relative to T0 of −2.2 in liquid xylose media and −4.1 in solid xylose media) is predicted to encode for a mannosyl-transferase involved in protein glycosylation, TRIATDRAFT_83791 (fitness relative to T0 of −5.1 in liquid xylose media and −2.0 in solid xylose media) is predicted to encode a major facilitator superfamily transporter, and TRIATDRAFT_238649 (fitness relative to T0 of −3.1 in liquid xylose media and −1.5 in solid xylose media) is predicted to encode for a peroxisome assembly factor (Fig. 5 *A* and *B* and Dataset S5).

Many of the genes in the xylose utilization pathway are well conserved in filamentous fungi (37). Therefore, we used mutants from the *Neurospora crassa* deletion collection (38, 39) to assess whether deletion mutants of the homologs of *xlnR* (*xlr-1*; NCU06971) and xylitol dehydrogenase (*xyd-1*; NCU00891) affected growth on xylose or the plant cell wall component xylan, which is primarily composed of polymers of β-1,4-linked xylose molecules. Cells lacking *xlr-1* were unable to grow on xylose or xylan, as previously demonstrated (34, 37) (*SI Appendix*, Fig. S15 and Dataset S6). As expected, a *xyd-1* deletion eliminated growth on xylose and significantly reduced growth on xylan (34, 36, 37) (*SI Appendix*, Fig. S15 and Dataset S6).

Only five additional genes with a xylose-specific fitness phenotype had potential predicted roles in carbon metabolism. All five only had significant phenotypes in liquid xylose media (Fig. 5 *A* and *C* and Dataset S5). Two of these genes have predicted roles in carbohydrate metabolism. The predicted galactonate dehydratase TRIATDRAFT_32165 had a fitness relative to T0 of −3.0 in liquid xylose media, while the glycoside hydrolase family 18 member TRIATDRAFT_83999 had a fitness relative to T0 of −2.7 in liquid xylose media. Three other genes have predicted roles in lipid or fatty acid metabolism with unclear roles in carbohydrate metabolism: The predicted choline dehydrogenase gene TRIATDRAFT_302655 had a fitness relative to T0 of −5.1 on liquid xylose media, the predicted epoxide hydrolase gene TRIATDRAFT_319890 had a fitness relative to T0 of −1.8 on liquid xylose media, and the predicted arylacetamide deacetylase gene TRIATDRAFT_90421 had fitness relative to T0 of −3.0 on liquid xylose media (Fig. 5 *A* and *C* and Dataset S5).

Genes with roles in transcriptional regulation and signal transduction also had xylose-specific fitness phenotypes in liquid media. A gene predicted to encode a mitogen-activated protein kinase in the hyperosmotic response pathway (TRIATDRAFT_157499) had a fitness relative to T0 of −8.4 in liquid xylose media, and a gene predicted to encode a member of a histidine kinase pathway (TRIATDRAFT_149807) had a fitness relative to T0 of −4.7 on liquid xylose media. Two genes predicted to encode transcription factors aside from *xlnR* had reduced fitness in liquid xylose media: TRIATDRAFT_92406 (fitness relative to T0 of −4.4 on liquid xylose media and −9.3 on solid xylose media) and TRIATDRAFT_191764 (fitness relative to T0 of −3.7 on liquid xylose media). Additionally, a predicted histone acetyltransferase gene (TRIATDRAFT_223094) had a fitness relative to T0 of −6.2 on xylose liquid media (Fig. 5 *A* and *C* and Dataset S5).

### Correlation Between Genes Required for Growth on Xylose and Genes Activated in Response to Xylan.

Many genes necessary for alternative carbon source utilization are activated in the conditions in which they are necessary (40). We hypothesized gene fitness would correlate with genes highly expressed during exposure to a similar carbon source, as seen in *R. toruloides* (41). To test our hypothesis, we exposed wild type *T. atroviride* to xylan (primarily composed of β-1,4-linked xylose monomers), glucose, and carbon starvation and used RNA sequencing (RNAseq) to measure gene expression. Genes with predicted roles in xylose utilization, including a predicted xylose reductase (TRIATDRAFT_259849), a predicted xylose dehydrogenase (TRIATDRAFT_303042), a predicted xylulokinase (TRIATDRAFT_219413), and a predicted xylose transporter (TRIATDRAFT_44225) were upregulated by more than ninefold on xylan as compared to carbon starvation and more than 38-fold on xylan as compared to glucose (Dataset S7).

As seen in bacterial species (42), we saw little correlation between gene fitness data on xylose and gene expression data on xylan (*SI Appendix*, Fig. S16). Three hundred fifty-four genes were at least fourfold differentially expressed in wild type cells during exposure to xylan compared to carbon starvation, and 595 genes were at least fourfold differentially expressed in wild type cells during exposure to xylan compared to glucose conditions (Dataset S7). Only two genes with a xylose specific fitness phenotype were also differentially expressed by at least fourfold during exposure to xylan compared to carbon starvation and glucose conditions (*SI Appendix*, Fig. S16 and Dataset S7): a predicted xylitol dehydrogenase gene TRIATDRAFT_303042 and a predicted galactonate dehydratase TRIATDRAFT_32165. In addition, TRIATDRAFT_302132, which encodes a hypothetical protein, was upregulated by 21.9-fold on xylan compared to carbon starvation. Mutations in TRIATDRAFT_302132 resulted in a fitness relative to T0 of −3.7 in liquid xylose media but did not have enough counts to be assigned a fitness score in solid xylose media (Fig. 5*C* and Dataset S7).

Four additional genes with a xylose-specific fitness phenotype were differentially expressed by at least fourfold during exposure to xylan compared to glucose (*SI Appendix*, Fig. S16*B* and Dataset S7). These genes included the transcription factor *xlnR* (TRIATDRAFT_78601), TRIATDRAFT_52267, which is predicted to encode an L-fuculose-phosphate aldolase (fitness relative to T0 of −1.7 on liquid xylose media), a predicted transcription factor gene TRIATDRAFT_92406 (fitness relative to T0 of −4.4 on liquid xylose media and −9.3 on solid xylose media) and a hypothetical protein encoding gene (TRIATDRAFT_ 301865; fitness relative to T0 of −5.2 on liquid xylose media) (Fig. 5*C* and Dataset S7).

## Discussion

The rapid increase in sequenced filamentous fungal genomes highlights the challenges of accurate gene annotation (43). This issue stems largely from difficulties in generating comprehensive, genome-wide phenotypic datasets for filamentous fungi. Classical forward genetic screens of randomly generated mutants or reverse genetic screens of filamentous fungal deletion mutant collections (38, 39, 44) are critical but are time-consuming tools to construct and/or utilize for functional gene characterization. A small number of targeted full or partial fungal deletion collections exist in well-studied filamentous fungi (38, 39, 44), one of which was used to identify a potential target for antifungal drugs to treat aspergillosis through a pooled fitness assay (45). However, these resources are prohibitively resource and time expensive for the many filamentous fungi with small research communities.

Here, we describe methods to establish RB-TDNAseq for high-throughput functional genomics in the filamentous fungus *T. atroviride*. In previous studies, ATMT and insertional mutagenesis were used to identify genes in a few filamentous fungi, including *Trichoderma reesei* and the plant pathogen *Magnaporthe oryzae* (46, 47). However, prior applications of insertional mutagenesis in filamentous fungi have typically used a tailed-PCR approach to identify mutants, which is laborious and requires substantial sequencing depth (47, 48). Here, we adapted high-throughput screening methods used in yeast and bacteria (9–11) to generate a randomly barcoded insertional mutant library to enable high-throughput functional genomics in filamentous fungi. Our development of an *A. tumefaciens* plasmid library containing an estimated 290 million uniquely barcoded plasmids that can be used for transformation in a variety of fungi and containing a resistance marker (hygromycin B) that is widely used in filamentous fungi will greatly expedite functional analysis of filamentous fungal genomes (Dataset S1). In this study, we highlight variables in the ATMT protocol important for increasing transformation efficiencies in filamentous fungi, facilitating the development of both insertional mutant libraries and improved targeted transformation protocols in a variety of filamentous fungi.

RB-TDNAseq libraries enable inexpensive high-throughput screens (9–11). After an initial deep sequencing step to map insertion locations and association of insertions with a unique DNA barcode, pooled fitness screens can be performed by PCR using universal primers that flank the barcode, followed by DNA sequencing. Barcode sequencing requires lower sequencing depth than is typically necessary in an unbarcoded insertional mutant library. However, performing pooled fitness screens in filamentous fungi presents challenges when compared to uninucleate yeast species (11, 49), due to multinucleate, syncytial cells and the potential for asexual cell fusion between germinated spores and hyphae. Additionally, some filamentous fungi have multinucleate, heterokaryotic asexual spores, necessitating purification of homokaryotic progeny prior to performing fitness assays to avoid potential complementation of loss-of-function mutations. In this study, the uninucleate asexual spores of *T. atroviride* circumvented this issue; asexual fusion between mutant strains that may have occurred during fitness assays did not eliminate our ability to detect fitness differences. Strategies for recovering large numbers of uninucleate or homokaryotic transformants, as well as methods to reduce the frequency of asexual fusion between hyphae—and, thus, complementation of loss-of-function mutants—during fitness assays, will be important considerations when applying this technology to other fungal species.

We focused on a proof-of-principal approach to determine whether RB-TDNAseq could be applied to filamentous fungi, using *T. atroviride* as a model. We generated a library of 83,311 barcoded insertions, disrupting 5,331 of 11,863 predicted genes in the *T. atroviride* genome. Some barcoded insertions were presumably not recovered because they occurred in essential genes or caused strongly deleterious phenotypes, were selected against during the transformation process, or were in genomic regions inaccessible to *A. tumefaciens*-mediated insertions. We estimate ~500,000 to several million barcoded insertional mutants are likely necessary to saturate a library in a typical filamentous fungus.

Like initial tests of BarSeq in bacteria and yeast species (9–11), we exposed the *T. atroviride* library to conditions that would enable identification of genes involved in carbon source utilization and primary metabolite biosynthesis. We identified genes with predicted roles in amino acid, nucleic acid, and folic acid biosynthesis and in fructose and xylose metabolism, confirming the efficacy of BarSeq in *T. atroviride* (Figs. 3–5). Our BarSeq assays also identified fitness phenotypes for several genes during exposure to xylose, fructose, and media lacking amino acids that have not previously been associated with these processes (Figs. 3–5). We identified three genes with xylose-specific fitness defects in both the liquid and solid BarSeq assays without clear roles in xylose utilization: TRIATDRAFT_238649, TRIATDRAFT_83791, and TRIATDRAFT_38402. Interrogating these genes using in silico approaches (50, 51) showed that TRIATDRAFT_238649 showed similarity to peroxisomal ATPases, TRIATDRAFT_83791 was similar to major facilitator superfamily transporters, and TRIATDRAFT_38402 was similar to glycolipid 2-α-mannosyltransferases, all without an obvious connection to xylose utilization. Genetic evidence from BarSeq assays potentially linking these three genes to xylose utilization illustrates the strength of the unbiased RB-TDNAseq screening approach. It will be the role of future studies to determine whether these genes are, indeed, involved in these metabolic pathways.

To examine the extent of correlation between expression profiles and mutant fitness, we compared our BarSeq data to RNAseq of *T. atroviride* exposed to related carbon sources. We saw limited correlation between our BarSeq and RNAseq data, with only five of the 37 genes with xylose-specific fitness defects also showing a significant increase in expression on the plant cell wall component xylan (Fig. 5). These genes included two major players in xylose and xylan utilization: the transcription factor *xlnR* and a gene encoding a xylitol dehydrogenase (Fig. 5). As seen in *R. toruloides* (41), fitness phenotypes in BarSeq experiments combined with differential expression in RNAseq experiments can be a strong indication of roles for genes in a particular biological process. However, not all genes with a role in a biological process are transcriptionally regulated during that process, while other transcriptionally regulated genes may play redundant roles. Thus, as seen in other organisms (42), many genes may only have fitness phenotypes while others only change in expression level. We believe a combination of these high-throughput assays could be a powerful tool for high-throughput functional genomics in filamentous fungi.

We successfully identified genes associated with primary metabolite biosynthesis and xylose and fructose utilization (Figs. 3–5), but consistency between replicates was low, especially for assays performed in liquid media (*SI Appendix*, Figs. S7 and S10–S13). Some of these inconsistencies are likely due to differences in the biology of filamentous fungi versus unicellular microbes. A larger mutant library with multiple insertions in every nonessential gene could reduce noise from asexual fusion because it would be possible to only compute fitness scores for genes in which multiple barcoded insertions are detected in BarSeq assays. Additionally, the lack of cytokinesis in mycelial growth may result in more divergent mutant populations in the T0 and experimental aliquots than in unicellular microbes where cytokinesis during an initial grow-out of a library aliquot results in equivalent mutant abundances in these populations (9–11). To decrease noise from stochasticity in mutant abundance in T0 versus experimental populations and between replicates, it may be helpful to begin experiments with larger starting populations, sequence BarSeq samples more deeply, and perform more than three biological replicates per experiment.

Although we focused our analysis on insertions in coding regions in this study, our library was biased away from insertions in coding regions and toward insertions in promoter and intergenic regions (Fig. 2*D*). A recent study using transposon mutagenesis in *Cryptococcus neoformans* demonstrated that it is possible to assay functions of essential genes using insertions in promoter regions (49). Future studies in filamentous fungi could use this technique to investigate the roles of essential genes. While we do not know the role that most noncoding DNA plays in filamentous fungi, many long noncoding RNAs (lncRNAs) and microRNAs have been identified in filamentous fungi, and several have known functions (52). For example, noncoding regions play a role in sexual reproduction in *Fusarium* graminearum (53) and microRNA-like RNAs play a role in injury response in *T. atroviride* (54). Future work will be necessary to determine whether it will be possible to use barcoded fitness assays to functionally annotate noncoding regions.

We demonstrate a method and provide crucial tools for relatively inexpensive generation of a mutant library containing tens of thousands of mutants for pooled, high-throughput fitness assays applicable to filamentous fungi across the fungal kingdom. The barcoded mutant library we generated in this study paves the way for studying fungal responses to environmental stimuli, fungal development, fungal–host interactions, and interactions between fungi and other microbes. Indeed, our protocols and *A. tumefaciens* plasmid library already enabled generation of an RB-TDNAseq library in the thermophilic fungus *Thermothelomyces thermophilus*, which was used to identify genes associated with germination at high temperature (55). The ease of obtaining BarSeq data in species with mapped barcoded TDNA insertions enables the creation of large datasets that can be exploited for gene discovery and functional characterization. In the industrially relevant bacterium, *Pseudomonas* *putida*, the large number of BarSeq experiments enabled machine learning techniques to predict functional relationships between genes (56). The development of RB-TDNAseq in filamentous fungi, particularly in medically, agriculturally, and biotechnologically relevant species, promises to reveal unknown gene functionalities, advancing fundamental fungal biology, enabling new treatments for fungal diseases of humans, animals, and plants, and improving fungal biotechnology.

## Materials and Methods

### Strains and Media.

*T. atroviride* IMI 206040 was the wildtype strain for generation of RB-TDNAseq libraries. *A. tumefaciens* EHA 105 was employed for barcoded library construction. *N. crassa* FGSC 2489 was the wild type control (57) and ∆*xlr-1* (NCU06971; FGSC 11067) and ∆*xyd-1* (NCU00891; FGSC 16529) mutants were obtained from the *N. crassa* deletion collection (38, 39). For growth and reagent details see *SI Appendix, SI Materials and Methods*.

### Randomly Barcoded Plasmid Library Construction.

We generated a large pool of uniquely barcoded plasmids using an optimized Type IIS endonuclease (Golden Gate) cloning strategy (17) modified from Coradetti et al. (11) to generate hundreds of millions to billions of uniquely barcoded plasmids (Dataset S1). For details see *SI Appendix, SI Materials and Methods*.

### *A. tumefaciens* Transformation with the Barcoded Plasmid Library.

To generate a diverse plasmid library for *A. tumefaciens* mediated transformation with an estimated 290 million uniquely barcoded plasmids, we transformed the pooled *E. coli* barcoded plasmids into *A. tumefaciens* EHA 105 using a method we optimized for high-efficiency transformation from Mersereau et al. (58). For details see *SI Appendix, SI Materials and Methods*.

### *A. tumefaciens* Mediated Transformation of *T. atroviride*.

We performed *A. tumefaciens* mediated transformation of *T. atroviride* using a method we optimized for high-efficiency transformations of filamentous fungi from Bundock et al. (59), de Groot et al. (19), and Coradetti et al. (11). For details see *SI Appendix, SI Materials and Methods*.

### Genomic DNA Extraction from the *T. atroviride* Barcoded Insertional Mutant Library.

Genomic DNA was extracted from *T. atroviride* barcoded insertional mutant library conidia using the Quick-DNA™ Fungal/Bacterial Miniprep kit (Zymo Research; D6005). For details see *SI Appendix, SI Materials and Methods*.

### TDNAseq Library Preparation.

To link barcode sequences to their genomic locations in the barcoded insertional mutant library, we sequenced TDNA insertion sites (TDNAseq) by adapting a modified version of the KAPA DNA sequencing library preparation protocol (21) for accurate mapping of TDNA insertions in filamentous fungal genomes. For details see *SI Appendix, SI Materials and Methods*.

### Mapping TDNA Insertion Locations.

Barcoded TDNA insertion locations in the *T. atroviride* RB-TDNA library were mapped to the *T. atroviride* V2 genome using the GFF file available at the National Center for Biotechnology Information (NCBI) (https://www.ncbi.nlm.nih.gov/datasets/genome/GCA_000171015.2/) (22) with the default settings of the RBseq 1.1.9 pipeline (https://github.com/stcoradetti/RBseq) (11, 29). For details see *SI Appendix, SI Materials and Methods*.

### Fitness Assays in Liquid and Solid Media.

To perform fitness assays (BarSeq), we compared a starting (T0) population to a matched population after exposure to experimental conditions (minimal medium, complete medium, complete medium without cysteine, xylose, glucose, and fructose). For details see *SI Appendix, SI Materials and Methods*.

### BarSeq Library Preparation and Sequencing.

To assess the relative abundance of barcoded mutants in the mutant library, we sequenced the barcodes present in each population (BarSeq) using a protocol adapted from those for yeast and bacteria (9–11). For details see *SI Appendix, SI Materials and Methods*.

### Fitness Analysis.

Fitness scores were derived from BarSeq data as described (9, 11) using the RBseq 1.1.9 pipeline (https://github.com/stcoradetti/RBseq) (11, 29). For details see *SI Appendix, SI Materials and Methods*.

### *N. crassa* Growth Assays.

To assess the growth of *N. crassa* mutants containing deletions of orthologs of *T. atroviride* genes identified in the xylose BarSeq experiments, we measured biomass accumulated by strains from the *N. crassa* deletion collection (38, 39). For details see *SI Appendix, SI Materials and Methods*.

### RNA Sequencing and Analysis.

RNA was extracted from *T. atroviride* exposed to media containing either no carbon source, 1% glucose, or 1% beechwood xylan (Sigma). Illumina libraries were prepared using poly(A) selection and sequenced on an Illumina NovaSeq6000 at the QB3 facility at UC Berkeley. For details see *SI Appendix, SI Materials and Methods*.

## Supplementary Material

Appendix 01 (PDF)

Dataset S01 (XLSX)

Dataset S02 (XLSX)

Dataset S03 (XLSX)

Dataset S04 (XLSX)

Dataset S05 (XLSX)

Dataset S06 (XLSX)

Dataset S07 (XLSX)

Dataset S08 (XLSX)

## Data Availability

Raw TDNAseq and BarSeq data are available on the NCBI Sequence Read Archive (SRA) through accession number PRJNA1347553 (60). Raw RNAseq data are available on the NCBI Gene Expression Omnibus (GEO) through accession number GSE325093 (61). Processed TDNAseq data are available in Dataset S2. Processed BarSeq data are available in Datasets S1, S4, and S5. Processed RNAseq data are available in Dataset S7. Raw *N. crassa* growth data are available in Dataset S6.
