# Supplementary material for: Barcoded mutant library enables high-throughput functional genomics in a filamentous fungus
Source: Proc Natl Acad Sci U S A. 2026 Aug 25;123(35):e2616888123. doi: 10.1073/pnas.2616888123 (PMC13534542; doi:10.1073/pnas.2616888123)
Supplement: Supplementary file 1 [file pnas.2616888123.sapp.pdf]

PNAS

Supporting Information for

Barcoded mutant library enables high-throughput functional genomics in a filamentous fungus

Lori B. Huberman[1,2#], José M. Villalobos-Escobedo[2,3,4#*], Jeffrey M. Skerker[5], Ran Shi[2], Adriana M. Rico-Ramírez[2], Catharine A. Adams[2,3], Adam P. Arkin[3,5], Adam M. Deutschbauer[2,3], and N. Louise Glass[2,3*]

Corresponding authors
N. Louise Glass
Lglass@berkeley.edu

José M. Villalobos-Escobedo
jose.villalobos@tec.mx

This PDF file includes:
Supporting text
Figures S1 to S16
Legends for Datasets S1 to S8
SI References

Other supporting materials for this manuscript include the following:
Datasets S1 to S8

Supporting Information Text

SI MATERIALS and METHODS

Strains and media

Trichoderma atroviride IMI 206040 was used as the wild type strain for the generation of RB-TDNAseq libraries. *Agrobacterium tumefaciens* EHA 105 was employed for barcoded library construction. *Neurospora crassa mat A* wild type FGSC 2489 was used as the wild type control (1), and Δ*xlr-1* (NCU06971; FGSC 11067) and Δ*xyd-1* (NCU00891; FGSC 16529) deletion mutants were obtained from the *N. crassa* deletion collection (2, 3). For the growth of the mutant library, we used both Potato Dextrose Broth (PDB) and Potato Dextrose Agar (PDA) media (BD Difco™). Vogel's minimal media (VMM) (4) was used as a synthetic minimal medium and supplemented with 1% glucose, 1% fructose, 1% xylose, or 1% xylan (xylan from Beechwood, Sigma-Aldrich) as indicated. To supplement the media with amino acids, we added complete drop-out mix (USBiological, D9515) at a final concentration of 1.92 g/L. The complete drop-out mix contains alanine, arginine, asparagine, aspartic acid, cysteine, glutamic acid, glutamine, glycine, histidine, isoleucine, leucine, lysine, methionine, phenylalanine, proline, serine, threonine, tryptophan, tyrosine, valine, adenine, uracil, p-aminobenzoic acid, and inositol. The addition of the complete drop-out mix to PDA, PDB, or VMM is indicated with a plus sign, i.e., PDA+, PDB+, or VMM+.

Reagents and filtration supplies

Acetosyringone (CAS No. 2478-38-8), hygromycin B (CAS No. 31282-04-9), kanamycin (CAS No. 25389-94-0), and carbenicillin disodium salt (CAS No. 4800-94-6) were purchased from Millipore Sigma. For *A. tumefaciens*-mediated transformation, we used HAWP MF-Millipore membrane filters (0.45 µm pore size; HAWP04700). All filtration steps were performed with Thermo Scientific™ Nalgene™ Rapid-Flow™ sterile disposable bottle-top filters equipped with polyethersulfone (PES) membranes. All primers used in this study were purchased from IDT, and their sequences are listed in Dataset S8.

Randomly barcoded plasmid library construction

We generated a large pool of uniquely barcoded plasmids using an optimized Type IIS endonuclease (Golden Gate) cloning strategy (5) we modified from Coradetti *et al.* (6). The drug resistance cassette in the modified pGI2 (7) ATMT vector used in Coradetti *et al.* (6) (pDP11) was replaced with a hygromycin resistance cassette that expresses well in Sordariomycete species (Fig S1). A *Hin*dIII recognition site in the region between the two divergent *Sap*I/*Lgu*I recognition sites was added just inside the right border of the TDNA in the pDP11 vector. We used these two divergent *Sap*I/*Lgu*I recognition sites to introduce the random barcodes. A population of random barcodes was generated by synthesizing the oligonucleotide GATGTCCACGAGGTCTCTNNNNNNNNNNNNNNNNNNNNCGTACGCTGCAGGTCGAC and amplifying it using the primers TCACACAAGTTTGTACAAAAAAGCAGGCTGGAGCTCGGCTCTTCGCCCGATGTCCACGAGGTCTCT and CTCAACCACTTTGTACAAGAAAGCTGGGTGGATCCGCTCTTCAATTGTCGACCTGCAGCGTACG (6). We combined 4 µg of plasmid DNA with 140 ng of barcode PCR fragments in a 50 µl reaction with 5 µl 10x T4 ligase buffer (ThermoFisher), 2.5 µl T4 ligase (ThermoFisher), and 2.5 µl *Lgu*I (ThermoFisher). We incubated the reaction for 5 min at 37°C, then 25 cycles of 2 min at 37°C, followed by 5 min at 16°C. The enzymes were then heat inactivated for 10 min at 65°C. We let the product cool to 37°C, added 2.5 µl FastDigest Buffer (ThermoFisher), 2.5 µl Tango Buffer (ThermoFisher), 3.13 µl 2 mg/ml bovine serum albumin (NEB), 12.5 µl FastDigest *Hin*dIII (ThermoFisher), and 12.5 µl *Lgu*I (ThermoFisher), and incubated the reaction at 37°C for 5 h to digest any uncut vector, then cooled to 4°C. The reaction products were purified using the Zymo

DNA Clean and Concentrator-5 Kit (Zymo Research) and eluted into 15 µl of ddH_2O. To ensure a large diversity in the plasmid library, we pooled the products of 18 barcoded plasmid cloning reactions. We then transformed 25 µl of NEB 10-β electrocompetent *Escherichia coli* (NEB) with 5 µl of the Golden Gate cloning reaction product according to the manufacturer's instructions in 48 independent transformations and selected for transformed *E. coli* using 50 µg/ml kanamycin. We pooled all transformants and estimated the diversity of the barcoded vector pool by sequencing plasmid barcodes on an Illumina HiSeq 4000 as described in Coradetti *et al.* (6), except the primers we used included dual indexes to prevent "index swapping" on the Illumina HiSeq 4000 (8, 9). The true pool size was estimated by the relative proportion of barcodes with 1 or 2 counts as described in the script Multicodes.pl (10). We estimate our barcoded plasmid pool contains approximately 1 billion uniquely barcoded plasmids (Dataset S1).

Agrobacterium tumefaciens transformation with the barcoded plasmid library

To generate a suitably diverse library of plasmids for *A. tumefaciens* mediated transformation of *T. atroviride*, we transformed the pooled barcoded plasmids into *A. tumefaciens* EHA 105 using a method we modified from Mersereau, *et al.*, 1990 (11). To generate electrocompetent *A. tumefaciens*, we inoculated *A. tumefaciens* into 5 ml LB and incubated at 30°C with shaking at 250 rpm for 16 h. We then back diluted the *A. tumefaciens* culture into 5 ml LB and incubated at 30°C with shaking at 250 rpm for at least 2 doublings until the cultures were at an optical density 600 (OD600) of 1. We then back diluted a second time into 100 ml LB flasks such that they would be at OD600 of 1 after an overnight incubation at 30°C with shaking at 250 rpm. *A. tumefaciens* cultures at OD600 of 1 were chilled on ice with slow swirling for 45 min. These cultures were then decanted into ice-cold 50 ml conical tubes and spun at 4000 rpm for 15 min at 4°C. The cell pellet was then washed once in 50 ml ice-cold ddH_2O, once in 25 ml ice-cold ddH_2O, and once in 2 ml ice-cold 10% glycerol before resuspension in 150 µl of ice-cold 10% glycerol. Aliquots of 55 µl of cell suspension were then flash frozen in liquid nitrogen.

We transformed 153 aliquots of frozen cell suspensions. We thawed the frozen electrocompetent *A. tumefaciens* cells on ice and mixed the 55 µl cell suspension with 2 µg of barcoded plasmid DNA in ddH_2O. We then incubated the cell/DNA mixtures on ice for 2 min before transferring the cell/DNA mixtures to chilled 2 mm gap 96-well electroporation plates (BTX) and electroporating using a 12.5 kV/cm field strength exponential decay wave (electroporation settings: 2500 V, 25 µF, 400 ohms) on a Gemini X2 high throughput electroporation system (BTX). We then immediately transferred the electroporated cells to 1 ml of 30°C LB and gently suspended the cells prior to incubating the cultures for 2 h at 30°C with shaking at 250 rpm. Transformed *A. tumefaciens* cells were selected with 50 µg/ml kanamycin. We pooled all transformants and sequenced the barcodes of a subset of the pool to estimate the diversity of the barcoded plasmid pool as described above. We estimate the barcoded plasmid pool housed in *A. tumefaciens* contains 290 million uniquely barcoded plasmids (Dataset S1).

A. tumefaciens mediated transformation of T. atroviride

We performed *A. tumefaciens* mediated transformation of *T. atroviride* using a method we optimized from Bundock *et al*., 1995 (12), de Groot *et al*., 1998 (13), and Coradetti *et al.*, 2018 (6). We grew the barcoded *A. tumefaciens* pool to an OD600 of 1 in 50 ml of LB with 50 µg/ml kanamycin in a baffled flask at 30°C at 250 rpm overnight. We then centrifuged the cells, resuspended them in 10 ml of induction medium (ABI medium – described below), and incubated them for 24 h at room temperature in cultures tubes on a roller drum. To generate *T. atroviride* conidia, we cultured *T. atroviride* on PDA for 84 h at 28°C in constant light. After harvesting the conidia with sterile ddH_2O, $2x10^7$ *T. atroviride* conidia were pelleted by centrifugation at 4000 rpm for 15 min, and the supernatant was discarded. The pellet was then

resuspended in 2 ml of the induced *A. tumefaciens* culture at an OD600 of 1 and incubated for 5 min at room temperature. The *T. atroviride*-*A. tumefaciens* mixed culture was then filtered onto a 0.45 µm pore membrane filter with a 47 mm diameter. For our library construction, we used a total of 100 filters to transform ~$2x10^9$ *T. atroviride* conidia. Filters with *T. atroviride-A. tumefaciens* co-cultured cells were transferred onto petri plates containing ABI medium with 2% Bacto agar with the cells facing up and incubated at 21°C for 7 d in darkness.

To induce *A. tumefaciens*-mediated transformation of *T. atroviride* conidia, we prepared a modified co-cultivation medium (ABI medium) based on AB minimal medium (6, 14). The ABI medium is composed of several stock solutions: 50 ml/L 20x AB salts solution, 2.4 ml/L 500 mM phosphate buffer at pH 7.5, 500 ml/L 2x 2-(N-morpholino)ethanesulfonic acid (MES) buffer at pH 5.0, and 1 ml/L 500 mM acetosyringone solution (added immediately prior to use). This solution was supplemented with 0.2% glucose (w/v), 0.05% casamino acids (w/v), and 0.001% thiamine (w/v) and stored at 4°C until needed. The 20x AB salts contained 20 g/L ammonium chloride (NH_4Cl), 6 g/L magnesium sulfate heptahydrate ($MgSO_4 \cdot 7H_2O$), 3 g/L potassium chloride (KCl), 0.2 g/L calcium chloride ($CaCl_2$), and 15 mg/L iron (II) sulfate heptahydrate ($FeSO_4 \cdot 7H_2O$) and was filter sterilized. The 500 mM phosphate buffer contained 60 g/L dipotassium phosphate (K_2HPO_4) and 20 g/L sodium dihydrogen phosphate (NaH_2PO_4), with pH adjusted to 7.5 using 2 M KOH. The 2x MES buffer contained 9.72 g/L MES, with pH adjusted to 5.0 with 2 M KOH. The 500 mM acetosyringone solution contained 0.098 g/ml acetosyringone in dimethyl sulfoxide (DMSO) and was either freshly prepared or aliquoted and stored at -20°C.

After a 7 d co-cultivation of *T. atroviride* conidia with *A. tumefaciens* on filters on ABI solid medium, we harvested the transformed *T. atroviride* conidia by vigorously vortexing each 0.45 µm membrane filter in 5 ml of sterile ddH_2O to detach the cells. We transferred 2 ml of the cell suspension to a sterile 2 ml microcentrifuge tube and centrifuged at 10,000 rpm for 5 min. The supernatant was carefully discarded, and the remaining suspension was added to the same tube and subjected to a second round of centrifugation under the same conditions. After discarding the final supernatant, the resulting pellet was resuspended in 1 ml sterile ddH_2O.

To select for transformed *T. atroviride* cells without contamination by untransformed *T. atroviride* cells or *A. tumefaciens* cells, we used a selection system consisting of multiple layers of agar in a 150 mm petri dish (Fig S2). We first poured a layer of 50 ml PDA + 200 µg/ml hygromycin B (to select for transformed *T. atroviride*) + 100 µg/ml carbenicillin (to select against growth of *A. tumefaciens*) and allowed it to solidify. A 13.5 ml PDA + 100 µg/ml carbenicillin layer was then poured over the first solidified layer. Once the second layer solidified, we then spread 10 ml of a suspension of conidia harvested from 10 filters on top of the second layer of agar. After the conidial suspension fully dried, we poured 13.5 ml of 37°C low-melting point PDA+ + 100 µg/ml carbenicillin over the conidial suspension. Once the low-melting point PDA+ solidified, we poured 13.5 ml of 55°C PDA+ + 100 µg/ml hygromycin + 100 µg/ml carbenicillin as the final layer to preserve selective pressure on *T. atroviride* (Fig S2). Inoculated plates were incubated at 28°C for 6 d in constant light to induce conidiation. Only fungal colonies capable of growing invasively through the top two layers of agar were recovered for downstream analysis.

Transformed *T. atroviride* conidia were harvested and resuspended in 10 ml ddH_2O. The suspensions were pooled and 50 ml aliquots were transferred to 50 ml conical tubes and centrifuged at 4100 rpm for 15 min to pellet the conidia. Each pellet was then resuspended in 10 ml sterile 30% glycerol (v/v) and stored at -80°C for long-term preservation. Because we expect the library to lose low-abundance and slow-growing mutants with each passage of the library, it is important to limit passaging the library as much as experimentally possible by generating many frozen aliquots in the initial library preparation to limit the need for library propagation. We collected transformants from ten 150 mm petri dishes, corresponding to the output of 100 *A. tumefaciens* mediated transformations. The protocol is available at: https://www.protocols.io/view/transformation-protocol-atmt-trichoderma-atrovirid-261gekk87g47/v1.

Genomic DNA extraction from the T. atroviride barcoded insertional mutant library

Genomic DNA was extracted from *T. atroviride* barcoded insertional mutant library conidia using the Quick-DNA™ Fungal/Bacterial Miniprep kit (Zymo Research; D6005). 10^9 conidia in 200 µl ddH2O were added to 500 µl Genomic Lysis Buffer. The conidial suspension was transferred to a ZR BashingBead™ Lysis Tube, followed by the addition of 750 µl BashingBead™ Buffer. The tubes were placed on ice and then processed in a Mini-Beadbeater-96 at maximum speed for 2 min. The remaining steps were carried out following the manufacturer's instructions. To assess DNA integrity, we ran the genomic DNA on a 1% agarose gel and looked for a single band without schmearing. To assess DNA purity, we quantified the DNA using both a NanoDrop spectrophotometer, which detects all nucleic acids, and the Qubit dsDNA High Sensitivity assay on a Qubit fluorometer to detect DNA specifically, to confirm the DNA did not have RNA contamination.

TDNAseq library preparation

To link barcode sequences to their genomic locations in the barcoded insertional mutant library, we sequenced the sites of TDNA insertions (TDNAseq) by adapting a modified version of the KAPA DNA sequencing library preparation protocol (15) for accurate mapping of TDNA insertions in filamentous fungal genomes. Briefly, 1 µg of high-quality genomic DNA was sheared to an average size of 300 bp using a Covaris sonicator compatible with microTUBE AFA Fiber Crimp-CAP tubes (6x16 mm; Covaris, cat. No. 520052 or 520091) in the presence of pH 8 TE buffer. Fragmented DNA was size-selected through a double-sided solid-phase reversible immobilization (SPRI) procedure using AMPure XP magnetic beads (Beckman Coulter, cat. no. A63881) and freshly prepared 80% ethanol for wash steps. The resulting DNA fragments underwent end-repair and A-tailing using the KAPA Hyper Prep Kit (Roche, cat. no. KK8502) according to the manufacturer's instructions. Adapter ligation was performed in the same reaction tubes using pre-annealed splinkerette adapters and ligation reagents included in the KAPA kit. The ligation products were purified using a single-sided SPRI bead cleanup.

Amplification of the adapter-ligated DNA was carried out in two successive PCR steps using KAPA HiFi HotStart ReadyMix (Roche, cat. no. KK2601). The first PCR selectively amplified fragments containing TDNA-genome junctions, and the product was cleaned using a double-sided SPRI selection to remove PCR products of the incorrect length. A second PCR was then performed to incorporate unique Illumina-compatible index sequences into each amplicon and select for products containing a TDNA insertion. This second PCR was followed by another double-sided SPRI cleanup to eliminate adapter dimers and PCR products of the incorrect length. The final library quality and fragment size distribution were assessed using an Agilent 2100 Bioanalyzer with the DNA 1000 kit (Agilent Technologies, cat. no. 5067-1504). Only libraries displaying a clear peak around 400 bp and minimal adapter contamination were sequenced. Sequencing was performed on an Illumina MiSeq at QB3 Genomics Sequencing Laboratory at University of California Berkeley (150 bp single-end reads) and on an Illumina NovaSeq6000 at Novagene (150 bp paired-end reads).

Mapping TDNA insertion locations

Barcoded TDNA insertion locations in the *T. atroviride* RB-TDNA library were mapped to the *T. atroviride* V2 genome using the GFF file available at the National Center for Biotechnology Information (NCBI) (https://www.ncbi.nlm.nih.gov/datasets/genome/GCA_000171015.2/) (16) using default settings in the RBseq 1.1.9 pipeline (https://github.com/stcoradetti/RBseq) (6, 17). TDNA insertions in samples sequenced with single end reads were mapped without any prior processing. We merged paired end reads using the default settings in BBMerge from BBMap 39.10 (18). We then mapped TDNA insertions using both the merged output files and the read 1 unmerged

output files. As described previously (6, 17), to filter true barcodes present in our library from sequencing errors, we eliminated any barcodes that differed from another, much more abundant, barcode within a Levenshtein edit distance of five and barcodes whose insertion mapped to more than one location in the *T. atroviride* genome, suggesting the TDNA had been inserted into multiple locations in one or multiple transformants.

Fitness assays in liquid and solid media

To perform fitness assays (BarSeq), we compared a starting (T0) population to a matched population after exposure to experimental conditions or an experimental condition to a matched control condition. For solid media fitness assays, frozen stocks of the mutant library containing 10^8 conidia were used to inoculate four PDA+ plates and incubated for 84 h at 28°C in constant light to expand the library and generate conidia. These conidia were harvested and split into a T0 population and matched aliquots to inoculate experimental conditions. The 10^8 conidia in each T0 population were inoculated into a flask containing 100 ml of liquid PDB+ medium and incubated for 48 h at 28°C with shaking at 220 rpm. The resulting mycelia were harvested for genomic DNA extraction and BarSeq to determine relative mutant abundance in the starting population. The experimental conditions were performed by inoculating 10^8 conidia onto plates containing solid VMM + 1.5% Bacto agar with 1% glucose, 1% fructose, or 1% xylose or containing solid VMM + 1% glucose + 1.5% Bacto agar without any supplementation (minimal medium), solid VMM+ + 1% glucose + 1.5% Bacto agar (complete medium), or solid VMM+ without cysteine + 1% glucose + 1.5% Bacto agar (complete medium without cysteine). Plates were incubated for 84 h at 28°C in constant light to allow growth and conidiation. After incubation, conidia were collected, and genomic DNA was extracted for subsequent BarSeq to measure the relative abundance of mutants in the final populations. Experiments were performed with 3 or 4 biological replicates as indicated in Datasets S4 and S5.

For liquid fitness assays, 10^8 conidia were inoculated from barcoded mutant library frozen stocks onto four PDA+ plates and incubated for 84 h at 28°C in constant light to expand the library and generate conidia. The conidia harvested from these plates were split into a T0 population containing 10^8 conidia and matched aliquots of 10^8 conidia used to inoculate experimental conditions. Genomic DNA was extracted directly from the T0 population conidia, and BarSeq was performed to determine relative mutant abundance in the starting population. The experimental conditions were performed by inoculating 10^8 conidia into 2.5 ml liquid VMM with either 1% glucose, 1% fructose, or 1% xylose in 12-well plates and incubated at 28°C for 48 h with shaking at 220 rpm. After incubation, the resulting mycelial biomass was collected, genomic DNA was extracted, and BarSeq was performed to determine relative mutant abundance in the final population. Experiments were performed with 4 biological replicates for each carbon source.

BarSeq library preparation and sequencing

To assess the relative abundance of barcoded mutants in the mutant library, we sequenced the barcodes present in each population (BarSeq). Genomic DNA was extracted from the T0 and experimental populations using the Zymo QuickDNA Kit (Zymo Research; D6005) following the manufacturer's instructions. The resulting genomic DNA was suspended in DNA elution buffer (Zymo Research), and its concentration was measured using the Qubit dsDNA High Sensitivity Assay on a Qubit 4.0 fluorometer. Barcodes were amplified by PCR using primers that annealed immediately up and downstream of the unique barcode and contained a unique 6 bp TruSeq index (Dataset S8) (9, 10). Amplification was performed using Q5 DNA polymerase (NEB) with Q5 GC enhancer (NEB) and the standard Q5 reaction buffer (NEB), using the following thermal cycling conditions: denaturation at 98°C for 4 min, followed by 25 cycles of 98°C for 30 s, 55°C for 30 s, and 72°C for 30 s, and then a final extension at 72°C for 5 min.

Equal volumes (10 µl) from each individual barcode PCR reaction were pooled, and 50 µl of the combined product was purified using the DNA Clean & Concentrator-5 Kit (Zymo Research). The final pooled BarSeq library was eluted into 50 µl of nuclease-free water and quantified using the Qubit dsDNA High Sensitivity Assay Kit (ThermoFisher Scientific). The barcodes were sequenced on an Illumina NovaSeq6000 at the QB3 Genomics Sequencing Laboratory at University of California Berkeley.

Fitness analysis

Fitness scores were derived from BarSeq data by taking the average of the $\log_2$(fold change) in relative barcode abundance of all barcodes associated with a particular gene as described (6, 10) using the RBseq 1.1.9 pipeline (https://github.com/stcoradetti/RBseq) using the default settings with the following exceptions (6, 17). Due to the lower number of insertions per gene in the *T. atroviride* mutant library than in previous barcoded libraries in yeast and bacteria species, for all fitness assays, we used a more lenient threshold to compute a gene fitness than was previously applied (6, 17) by requiring only one insertion in a gene. For fitness experiments where the library was exposed to minimal medium, complete medium, complete medium without cysteine, xylose, and glucose, we used the standard settings of requiring at least 20 barcode counts across all insertions in a gene in the T0 and experimental condition to compute a gene fitness. For fitness experiments where the library was exposed to fructose, we allowed a more lenient threshold of at least 10 barcode counts across all insertions in a gene in the T0 and experimental condition in a gene to compute a gene fitness. We also computed the glucose fitness using this more lenient threshold of at least 10 barcode counts across all insertions in a gene in the T0 and experimental condition to compute gene fitness for glucose specifically for direct comparison with the fructose data.

We used multiple methods to calculate statistical significance of fitness scores (6, 10, 17). The first was a *t*-like test statistic, which we will refer to as a *t*-statistic, that estimates the reliability of fitness scores (10). (For readers interested in the mathematics used to calculate this *t*-like test statistic, see Wetmore *et al*, 2015 (10).) The second is a p-value calculated from the *t*-like test statistic, in which the *t*-like test statistic is treated as a true *t*-statistic after Benjamini-Hochberg multiple hypothesis correction (6, 17). This p-value can be treated as a false discovery rate.

To determine genes with fitness defects specific to conditions of interest, we required that genes have a fitness of less than -1.5 with a *t*-statistic less than -3 in that condition, which was previously used to denote significant fitness in fungal barcoded libraries (6). We also required that genes have fitness scores computed for both the condition of interest and the control condition. To eliminate pleiotropic mutants from our analysis, we computed fitness comparing the condition of interest with a control condition by running RBseq_Calculate_Fitness.py 1.1.9 (https://github.com/stcoradetti/RBseq) (6, 17) with the control condition as the T0 (i.e., complete medium was used as the T0 for minimal medium and complete medium without cysteine; glucose was used as the T0 for xylose and fructose). We used the RBseq_Calculate_Fitness.py 1.1.9 (https://github.com/stcoradetti/RBseq) (6, 17) default settings with the following exceptions. We once again required only one insertion per gene to compute a gene fitness. We required at least 20 barcode counts across all insertions in a gene in the control and experimental condition to compute a gene fitness for comparisons of minimal medium and complete medium without cysteine with complete medium and xylose with glucose. We required only 10 barcode counts across all insertions in a gene in the control and experimental condition to compute a gene fitness for comparisons of fructose with glucose. To designate genes as having a fitness defect specific to the condition of interest, that gene had to have fitness scores less than -1.5 with *t*-statistics < -3 in both the condition of interest relative to T0 and the condition of interest relative to the control condition.

N. crassa growth assays

To assess the growth of *N. crassa* mutants containing a deletion of a *T. atroviride* ortholog identified in the BarSeq experiments, we used strains from the *N. crassa* deletion collection (2, 3). Strains were grown from freezer stocks in VMM with 2% sucrose + 1.5% agar in slant tubes. Conidia were collected after incubating at 30ºC for 4 d in constant dark and 3 d in constant light. Growth assays were performed in deep-well 24-well plates with four replicates per strain by inoculating 10^8 conidia in 2.5 ml VMM with either 1% glucose, 1% fructose, 1% xylose, or 1% beechwood xylan (Sigma). Cultures were incubated at 30ºC with constant shaking (220 rpm) for 24 h. After 24 h, the biomass was harvested and weighed while wet. Statistical significance of differences in growth was evaluated using ANOVA followed by Tukey's test.

RNA sequencing and analysis

T. atroviride was grown on VMM + 1% glucose + 1.5% agar for 36 h at 28ºC in constant darkness. A plug of mycelium was taken using a sterile number 6 (11 mm) cork borer to inoculate 125 ml Erlenmeyer flasks containing 50 ml liquid VMM with 1% glucose as the carbon source and grown for 48 h at 28ºC in darkness with shaking at 220 rpm. After 48 h, the spherical mycelial mass was removed and washed three times with VMM lacking a carbon source. Mycelia were then transferred to flasks containing 50 ml of VMM with either no carbon source, 1% glucose, or 1% beechwood xylan (Sigma) and incubated for 12 h at 28ºC in darkness with shaking at 220 rpm. After 12 h, the mycelium was harvested, the culture medium was decanted from the flask, and the mycelial mat was collected by filtration with a 90 mm Whatman Grade 3 filter (Whatman, WH1003-090). The mycelial mat was flash frozen in liquid nitrogen and ground for RNA extraction with a mortar and pestle. Three biological replicates were performed for each condition.

Total RNA was extracted using TRIzol (Invitrogen) and purified using the RNeasy Plus kit (Qiagen) following the manufacturer's protocol. Libraries were prepared for sequencing using the TruSeq RNA Sample Preparation Kit v2 (Illumina) with poly(A) selection. Paired-end 150 bp sequencing was performed on an Illumina NovaSeq6000 at the QB3 Genomics Sequencing Laboratory at University of California Berkeley, with an average depth of 30 million reads per library.

Quality control was conducted using FastQC (https://www.bioinformatics.babraham.ac.uk/projects/fastqc/). Reads were trimmed for adapter and index sequences using Trimmomatic (19) using the following settings: ILLUMINACLIP:TruSeq3-PE.fa:2:30:10:2:keepBothReads (allowing up to two mismatches for adapter detection, a palindrome clip threshold of 30, a simple clip threshold of 10, and retention of both reads in a pair even if only one contains an adapter sequence), LEADING:3, TRAILING:3, and MINLEN:36 to remove low-quality bases from the ends and discard reads shorter than 36 base pairs. Transcripts per million (TPM) were quantified using Salmon version 1.10.0 (20) using the -l A and –validateMappings flags on the processed read 1 fastq files. Differential expression was determined using DESeq2 version 1.48.0 (21).

SI FIGURES

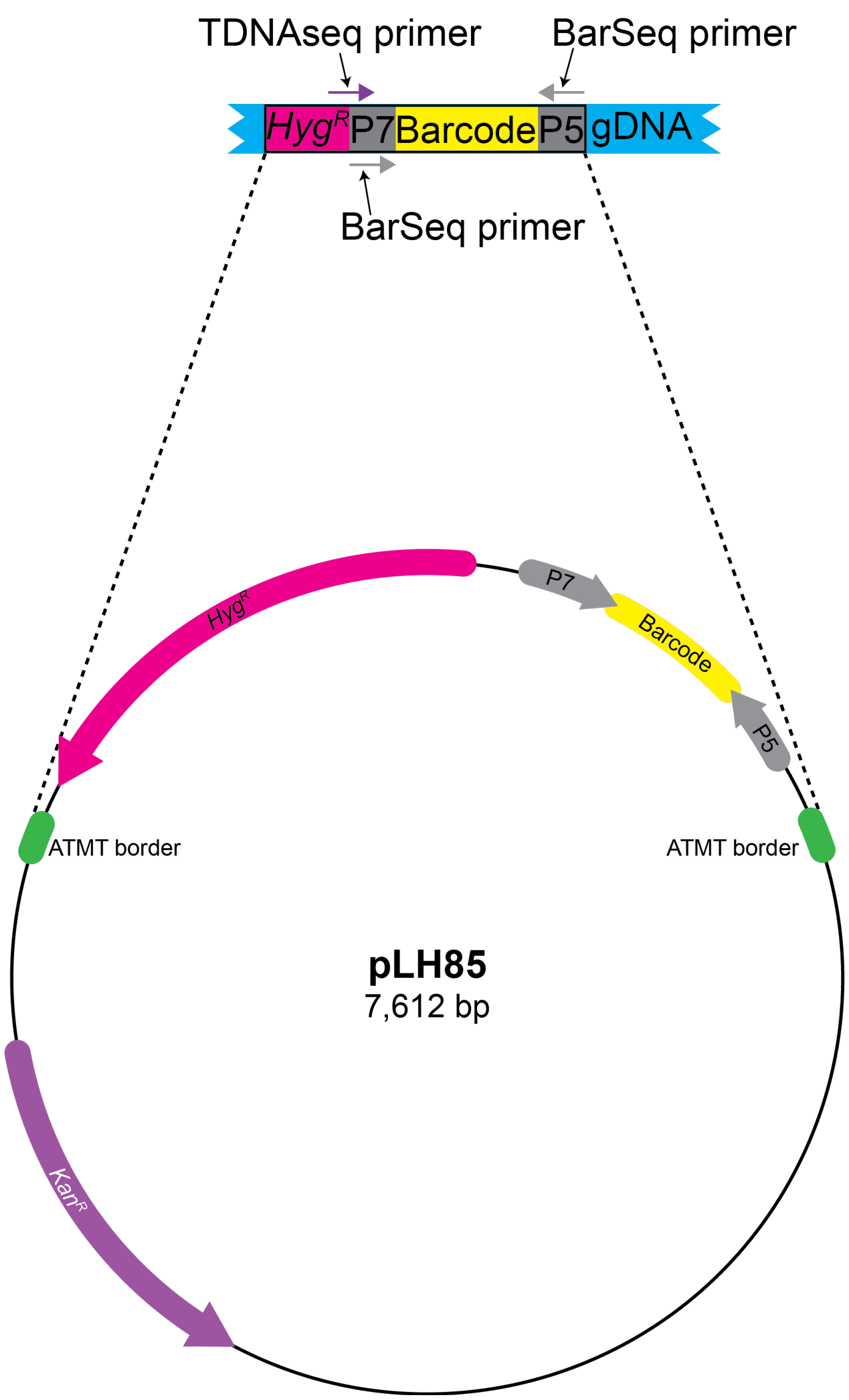

Fig S1. Circular map of the pLH85 plasmid for *Agrobacterium tumefaciens*-mediated transformation (ATMT) of *Trichoderma atroviride* with a randomly barcoded drug resistance cassette. The pLH85 plasmid is 7,612 bp and contains a kanamycin resistance gene (*Kan*^{R}) for selection in *E. coli* and *A. tumefaciens* (purple), a hygromycin resistance cassette (*Hyg*^{R}) for selection in fungi (pink), a 20 bp DNA barcode (Barcode; yellow), universal sequencing primers surrounding the barcode (P7 and P5; grey), and borders for ATMT insertion (green) (lower panel). The borders for ATMT indicate the region of the plasmid inserted as TDNA into the *T. atroviride* genome. The forward primer for TDNAseq is indicated in purple and the forward and reverse BarSeq primers for fitness assays are indicated in grey (upper panel). (Note: Cartoon is not drawn to scale.)

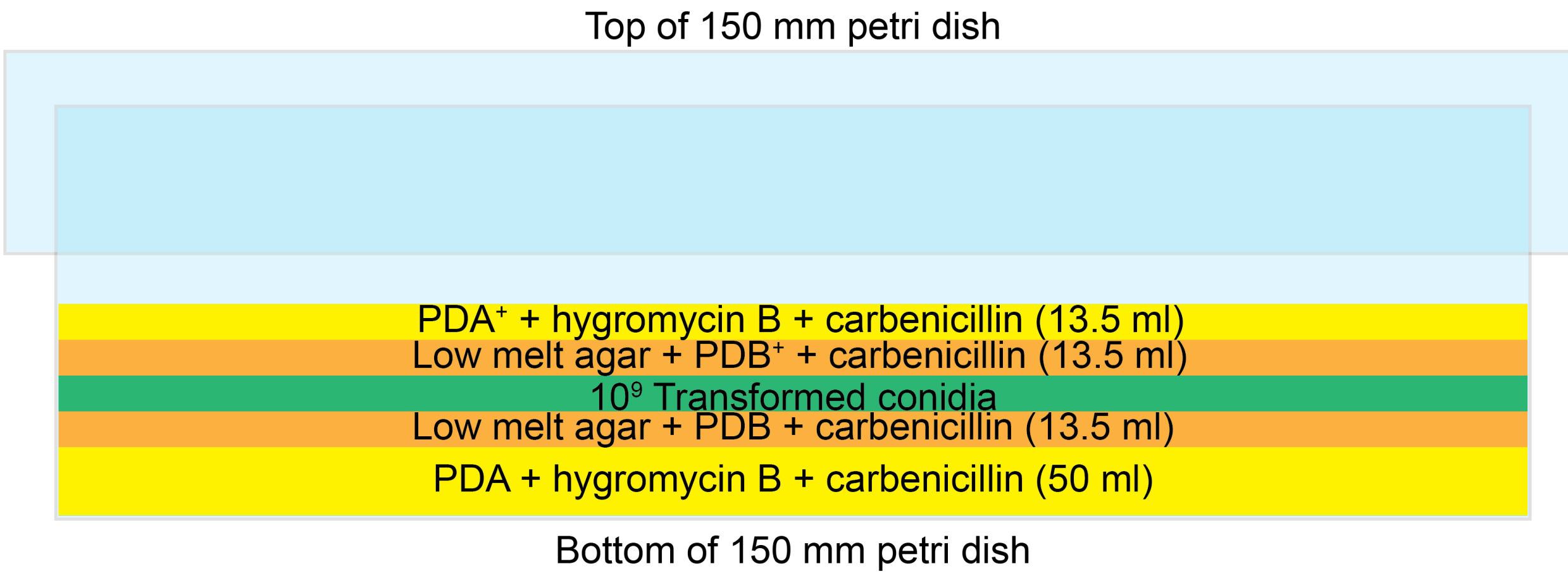

Fig S2. Schematic for recovering the transformed library. The indicated layers of media or conidial suspensions were poured sequentially into a 150 mm petri dish to separate transformed *T. atroviride* from *A. tumefaciens* after ATMT. Hygromycin B is used to select for transformed *T. atroviride*. Carbenicillin is used to select against the growth of *A. tumefaciens*. Each layer was allowed to fully solidify (agar layers) or dry (conidial layer) before the next layer was poured.

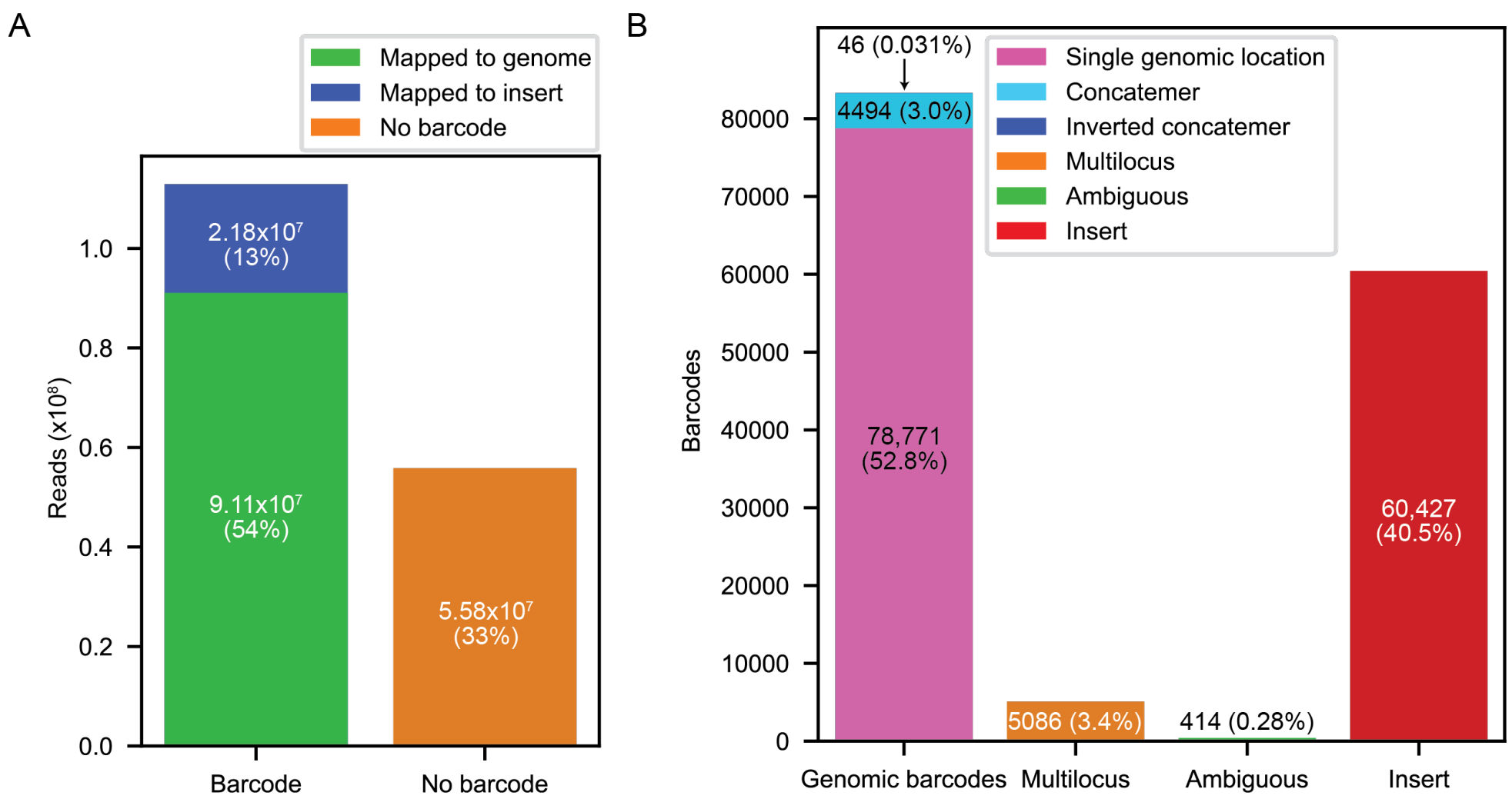

Fig S3. Distribution of reads and barcodes. (**A**) Distribution of reads with and without a barcode. Reads without a barcode are due to incorrect annealing of the amplification primers during TDNAseq library preparation to either the *T. atroviride* or *A. tumefaciens* genomes. Barcodes mapped to the insert (i.e., pLH85) are indicative of library contamination with *A. tumefaciens* containing unprocessed ATMT plasmids, incorrect processing of the plasmid during ATMT resulting in reads that map exclusively to the plasmid, or concatemer formation due to sequential insertions of multiple TDNAs. (**B**) Distribution of barcode mapping locations. Genomic barcodes are used for fitness assays and include single genomic location barcodes, concatemers, and inverted concatemers. Single genomic location barcodes mapped exclusively to one location in the genome. Concatemer barcodes indicate front-to-back insertions of the same TDNA insert, resulting in a barcode mapping both to another insert in the same direction and to the genome. Inverted concatemer barcodes indicate front-to-front inserts of TDNA resulting in a barcode mapping both to another insert in the opposite direction and to the genome. (Because there are only 46 instances of inverted concatemers, the dark blue bar is very thin at the top of the “Genome barcodes” stacked bar.) All other barcodes are not used for fitness assays and include: multilocus barcodes, ambiguous barcodes, and barcodes mapped to the insert. Multilocus barcodes mapped to more than one location in the genome. Ambiguous barcodes had genomic locations that could not be definitively determined due to reads into the genome that were too short, repetitive regions, etc. Insert barcodes mapped exclusively to the insert and did not also map to a genomic location, due to library contamination with *A. tumefaciens* containing unprocessed ATMT plasmids, incorrect processing of the plasmid during ATMT resulting in reads that map exclusively to the plasmid, or concatemer formation due to sequential insertions of multiple TDNA in which it was not possible to identify a final insertion that read directly into the genome.

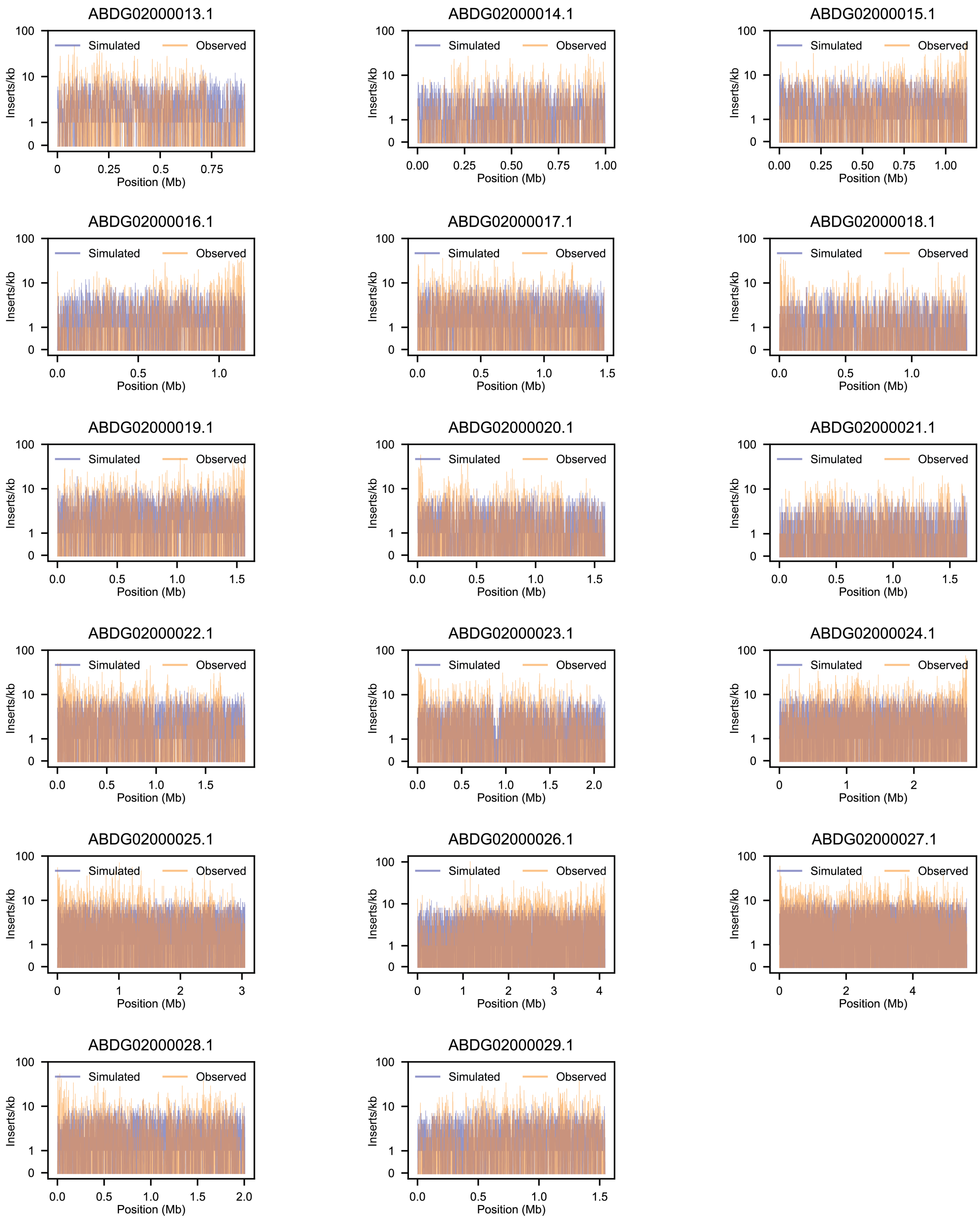

Fig S4. The 83,311 uniquely barcoded TDNA insertions are broadly distributed across the *T. atroviride* genome. Number of insertions per kb in 500 bp windows across the largest 17 scaffolds (16) (i.e., over 500 kb) (orange) compared to a simulation of random insertion density across each scaffold (blue).

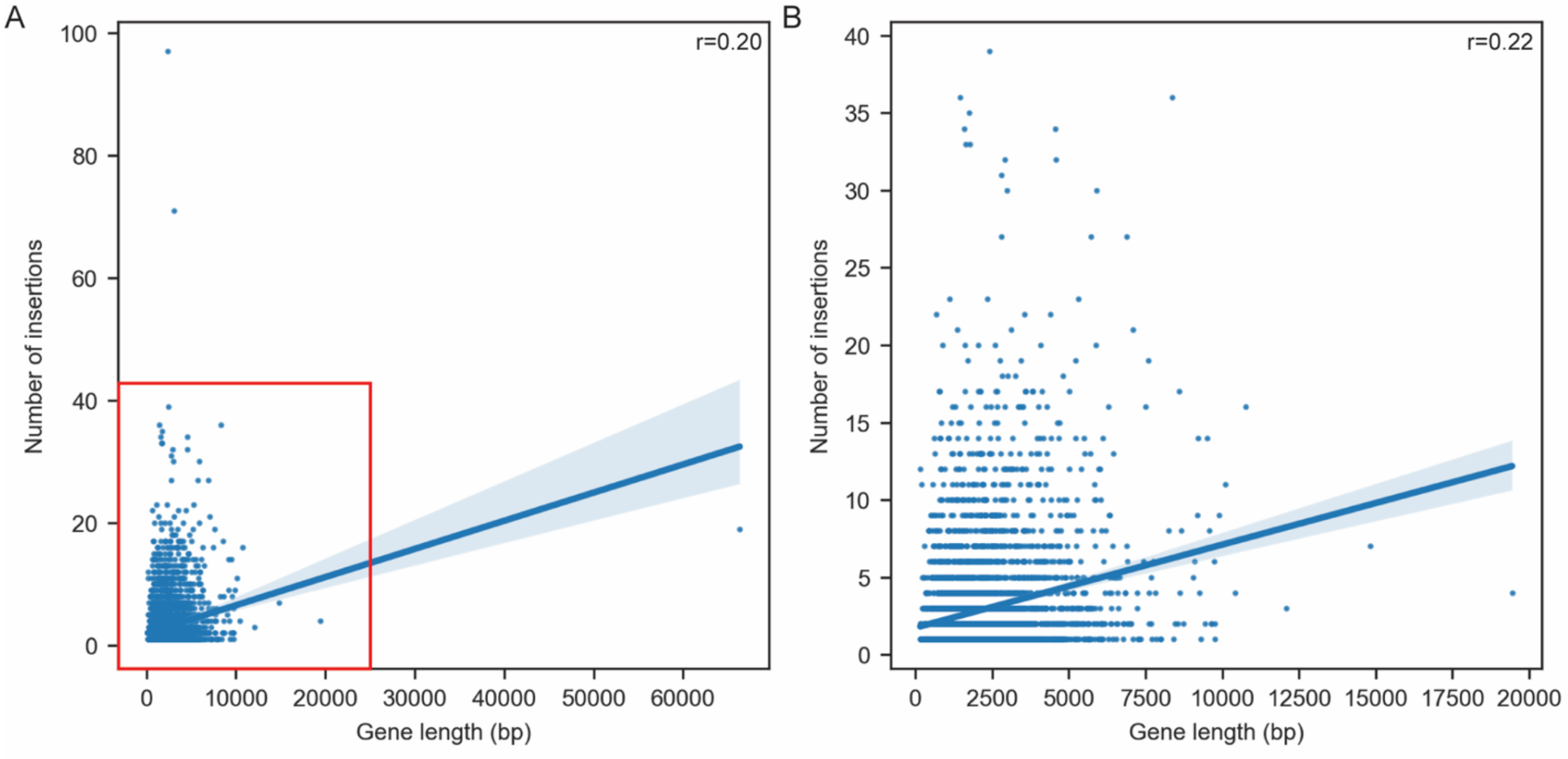

Fig S5. Number of insertions per gene does not correlate well with gene length. (**A-B**) Scatter plots of the number of insertions per gene relative to the length of the gene for (**A**) all genes with insertions or (**B**) all genes within the red box in (**A**). Lines are linear regression fits, and bands are 95% confidence intervals. R values for the linear regression lines are indicated on each graph ($p < 5x10^{-50}$).

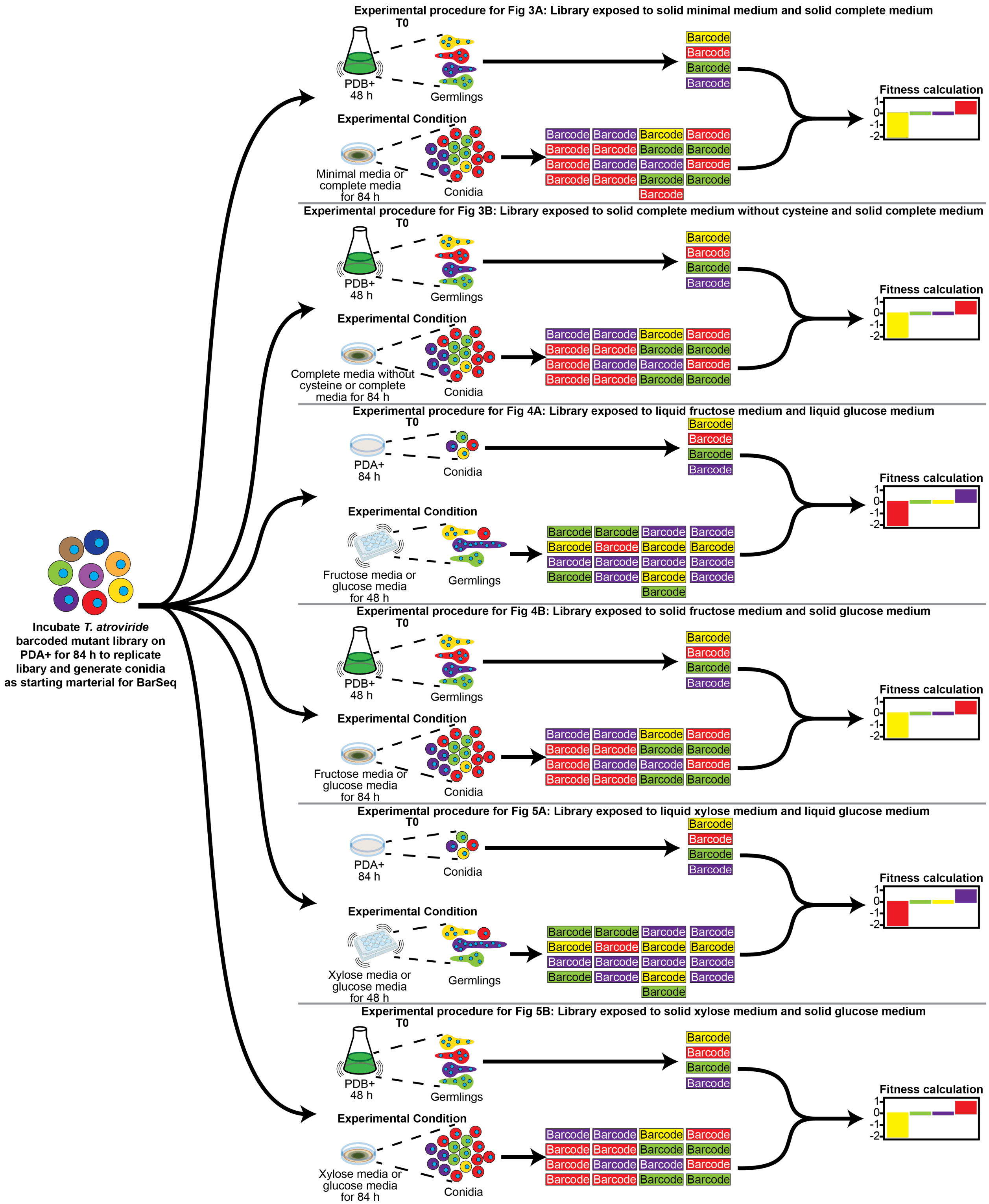

Fig S6. Schematic of BarSeq in *T. atroviride*. An aliquot of the barcoded mutant library was inoculated onto PDA+ plates and incubated for 84 h to replicate the library and generate conidia as starting material for BarSeq. These conidia were then used to inoculate paired T0 and experimental conditions for each of the BarSeq experiments. For the experiments shown in Fig

3A, 3B, 4B, and 5B, the conidia for the T0 were inoculated into PDB+ and incubated for 48 h to generate germlings. These germlings were then harvested to measure relative barcode abundance in the starting population. A paired population of conidia was inoculated onto complete media petri dishes (for Fig 3A and 3B), minimal media petri dishes (for Fig 3A), complete media without cysteine petri dishes (for Fig 3B), glucose media petri dishes (for Fig 4B and 5B), fructose media petri dishes (for Fig 4B), or xylose media petri dishes (for Fig 5B). These petri dishes were incubated for 84 h to generate conidia. These conidia were then harvested to measure relative barcode abundance in the experimental population. For the experiments shown in Fig 4A and 5A, the conidia for the T0 were inoculated onto PDA+ plates and incubated for 84 h to generate conidia. These conidia were then harvested to measure relative barcode abundance in the starting population. A paired population of conidia was inoculated into glucose liquid media (for Fig 4A and 5A), fructose liquid media (for Fig 4A), or xylose liquid media (for Fig 5A) in 12-well plates and incubated for 48 h to generate germlings. These germlings were then harvested to measure relative barcode abundance in the experimental population. To calculate fitness relative to the T0, the relative barcode abundance in the experimental population was compared to the relative barcode abundance in the paired T0 population. To calculate fitness relative to a control condition, the relative barcode abundance in minimal media or complete media without cysteine was compared to the relative barcode abundance in complete media (for Fig 3A and 3B); the relative barcode abundance in liquid fructose media or liquid xylose media was compared to the relative barcode abundance in liquid glucose media (Fig 4A and 5A); and the relative barcode abundance in solid fructose or solid xylose media was compared to the relative barcode abundance in solid glucose media (for Fig 4B and 5B).

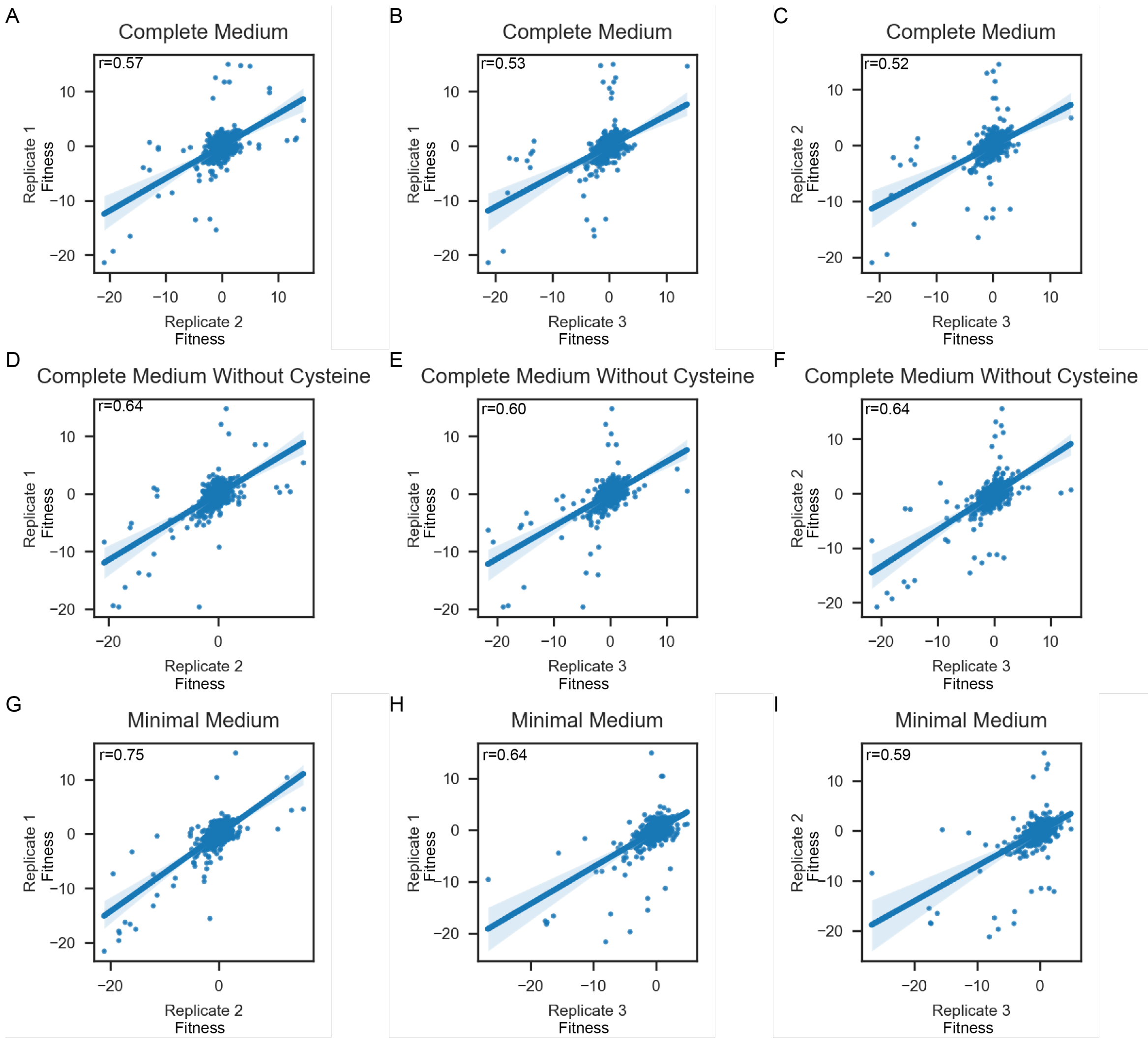

Fig S7. Comparison of BarSeq replicate gene fitness scores relative to T0 from the barcoded insertional mutant library grown on complete medium, complete medium without cysteine, and minimal medium. Comparison of gene fitness scores relative to T0 obtained from individual replicates of the insertional mutant library grown on (**A-C**) solid Vogel's minimal medium (VMM) (4) supplemented with amino acids, nucleic acids, and vitamins (complete medium), (**D-F**) solid VMM supplemented with nucleic acids, vitamins, and all amino acids except cysteine (complete medium without cysteine), or (**G-I**) solid VMM (minimal medium) for 84 h; conidia were subsequently harvested for BarSeq. Lines are linear regression fits, and bands are 95% confidence intervals. R values for the linear regression lines are indicated on each graph ($p < 10^{-193}$).

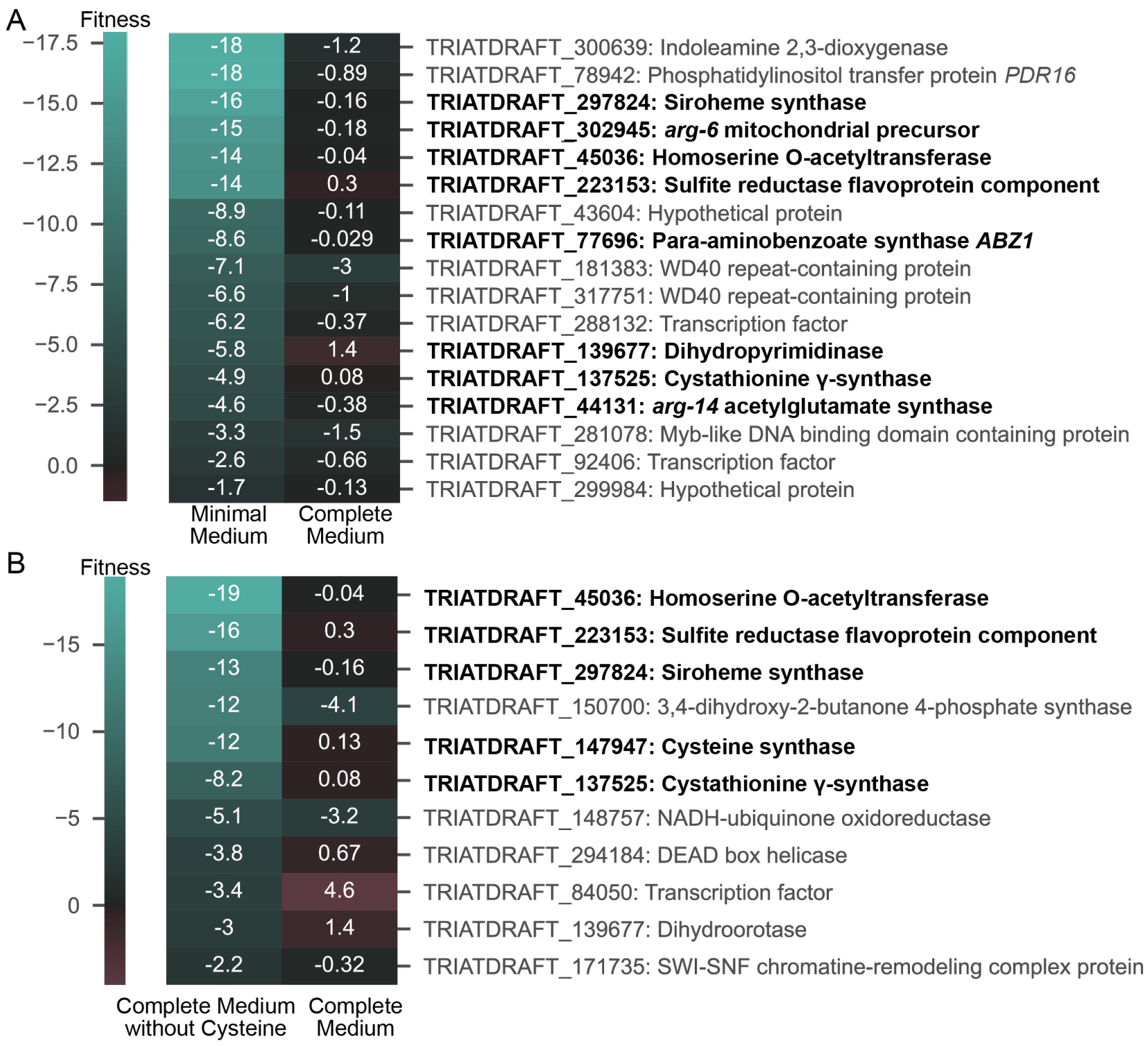

Fig S8. Identification of amino acid biosynthesis genes using BarSeq. (**A**) Heatmap of minimal medium relative to T0 fitness scores of genes with minimal medium relative to T0 and minimal medium relative to complete medium fitness scores below -1.5 with *t*-statistics < -3. Genes predicted to play a role in amino acid, nucleic acid, or folic acid biosynthesis are indicated in bold. Minimal medium and complete medium relative to T0 fitness scores are indicated on the heatmap. (**B**) Heatmap of complete medium without cysteine relative to T0 gene fitness scores of genes with complete medium without cysteine relative to T0 and complete medium without cysteine relative to complete medium fitness scores below -1.5 with *t*-statistics < -3. Genes predicted to play a role in sulfur amino acid biosynthesis are indicated in bold. Complete medium without cysteine fitness and complete medium fitness relative to T0 scores are indicated on the heatmap.

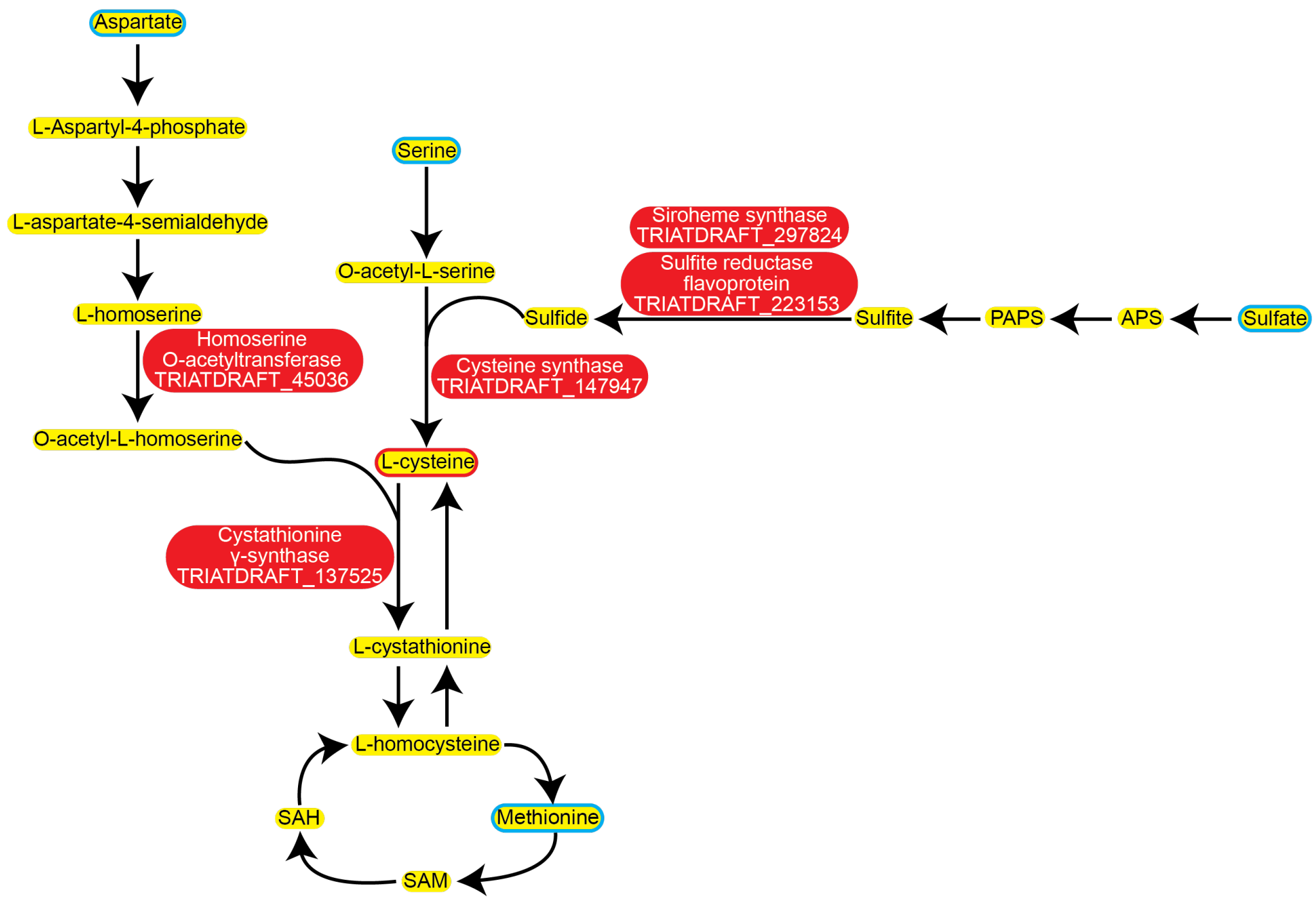

Fig S9. Sulfur amino acid biosynthesis pathway. Enzymes predicted to be encoded by genes with fitness phenotypes specific to complete media without cysteine in the BarSeq experiment (Fig 3B and Fig S8B) are indicated in red. Metabolites present in the complete medium are outlined in blue. Cysteine (missing from complete medium without cysteine) is outlined in red. (Abbreviations: APS = adenosine 5'-phosphosulfate; PAPS = 3'-phosphoadenosine-5'-phosphosulfate; SAM = S-adenosylmethionine; SAH = S-adenosylhomocysteine.)

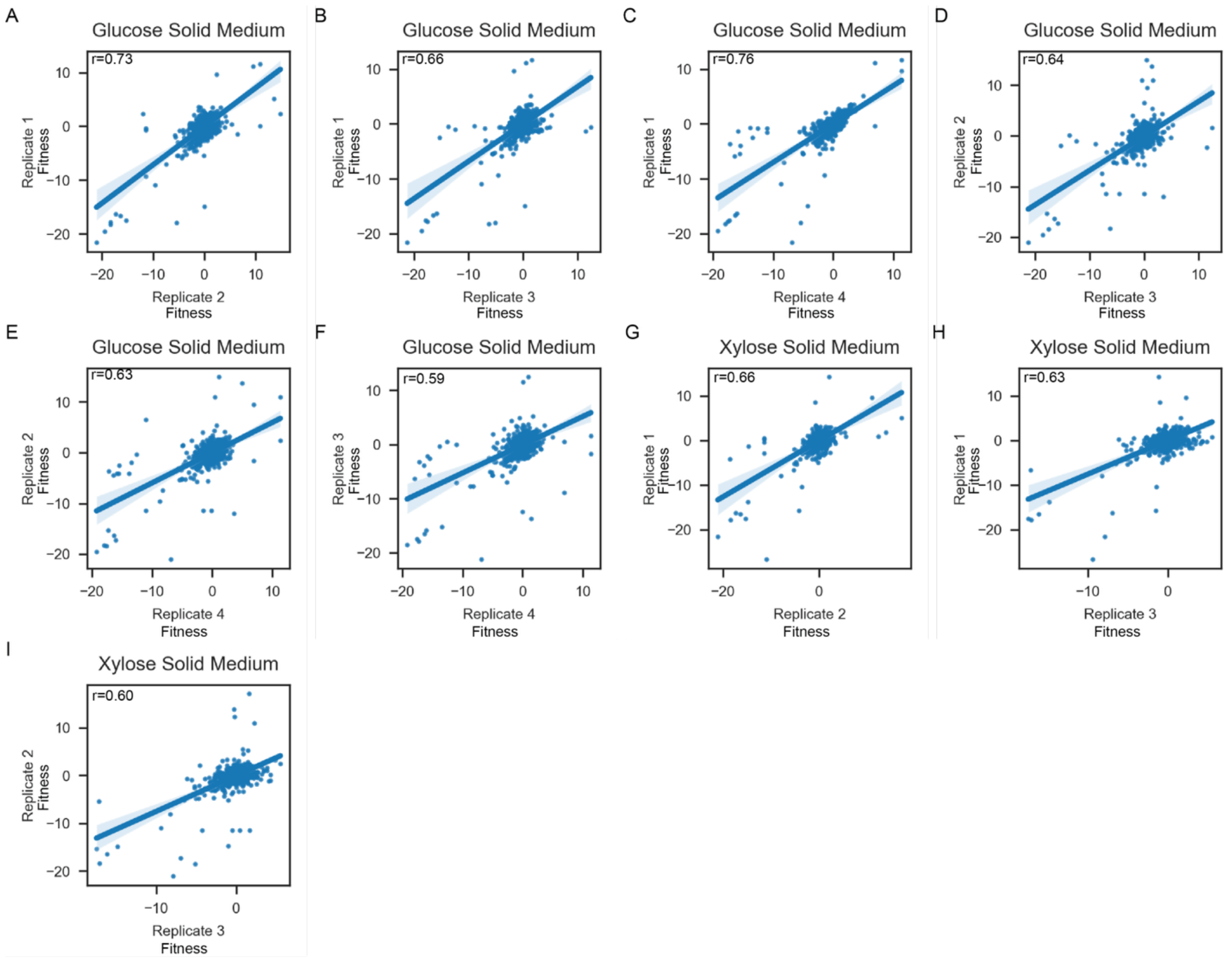

Fig S10. Comparison of BarSeq replicate gene fitness scores relative to T0 from the barcoded insertional mutant library grown on solid minimal medium containing glucose or xylose as the sole carbon source. Comparison of fitness scores relative to T0 obtained from individual replicates of the insertional mutant library grown on (**A-F**) solid minimal medium with glucose or (**G-I**) solid minimal medium with xylose for 84 h; conidia were subsequently harvested for BarSeq. Genes were included in the fitness analysis if there were at least 20 reads associated with insertions in the gene. Lines are linear regression fits, and bands are 95% confidence intervals. R values for the linear regression lines are indicated on each graph ($p < 10^{-264}$).

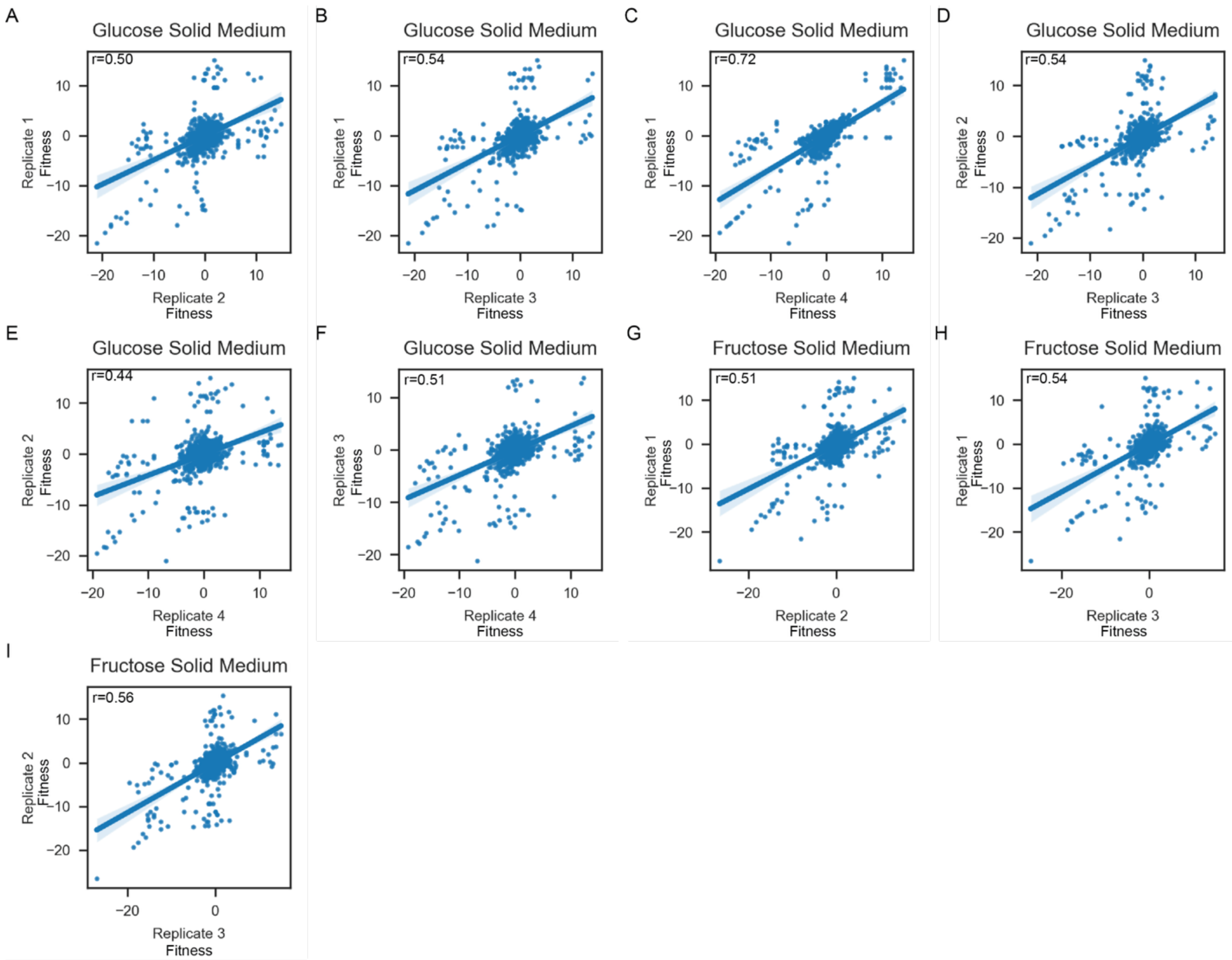

Fig S11. Comparison of BarSeq replicate gene fitness scores relative to T0 from the barcoded insertional mutant library grown on solid minimal medium containing glucose or fructose as the sole carbon source. Comparison of fitness scores relative to T0 obtained from individual replicates of the insertional mutant library grown on (**A-F**) solid minimal medium with glucose or (**G-I**) solid minimal medium with fructose for 84 h; conidia were subsequently harvested for BarSeq. Genes were included in the fitness analysis if there were at least 10 reads associated with insertions in the gene. Lines are linear regression fits, and bands are 95% confidence intervals. R values for the linear regression line are indicated on each graph ($p < 10^{-146}$).

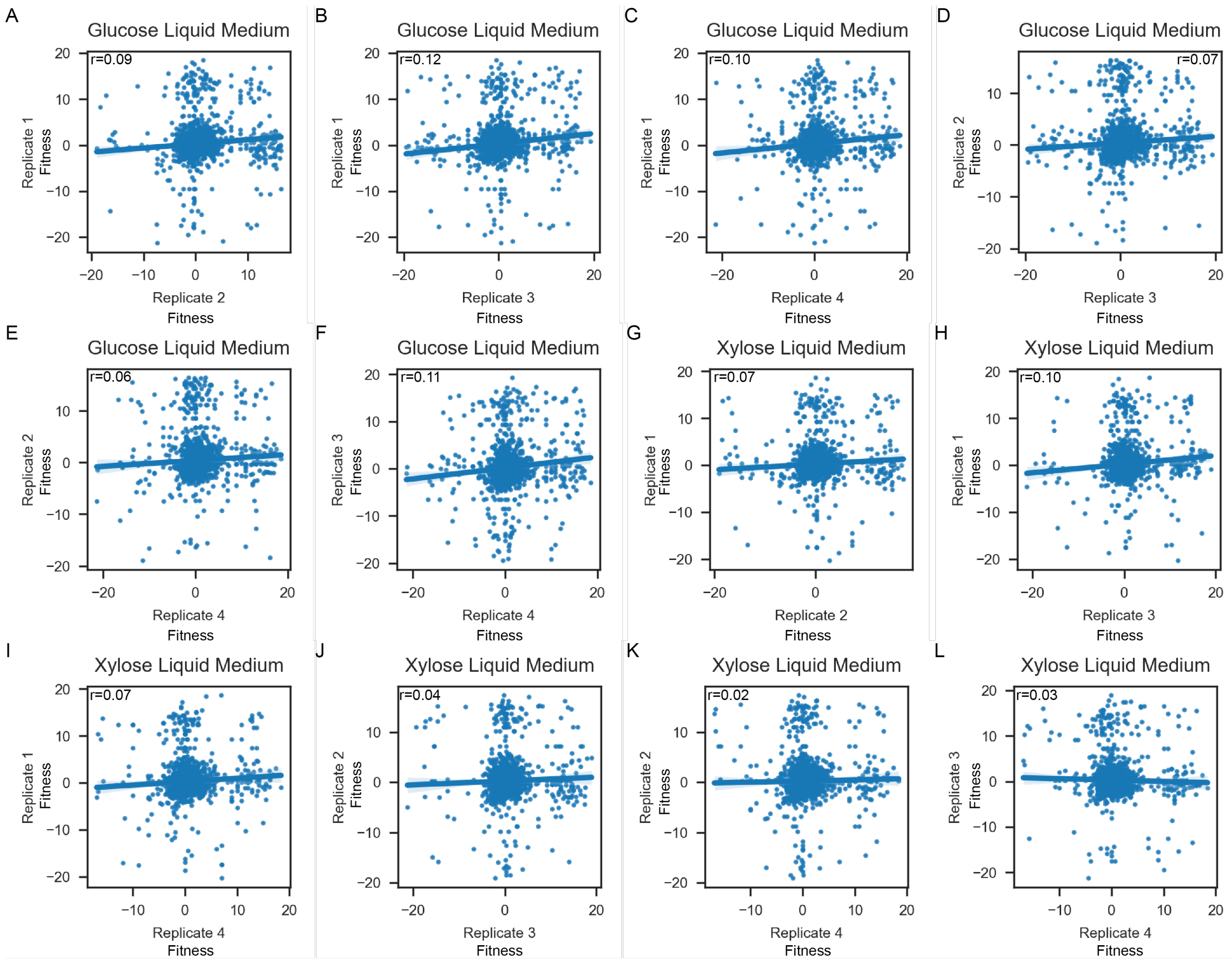

Fig S12. Comparison of BarSeq replicate gene fitness scores relative to T0 from the barcoded insertional mutant library grown on liquid minimal medium containing glucose or xylose as the sole carbon source. Comparison of fitness scores relative to T0 obtained from individual replicates of the insertional mutant library grown on (**A-F**) liquid minimal medium with glucose or (**G-L**) liquid minimal medium with xylose for 48 h; germlings were subsequently harvested for BarSeq. Genes were included in the fitness analysis if there were at least 20 reads associated with insertions in the gene. Lines are linear regression fits, and bands are 95% confidence intervals. R values for the linear regression lines are indicated on each graph ($p \leq 0.26$).

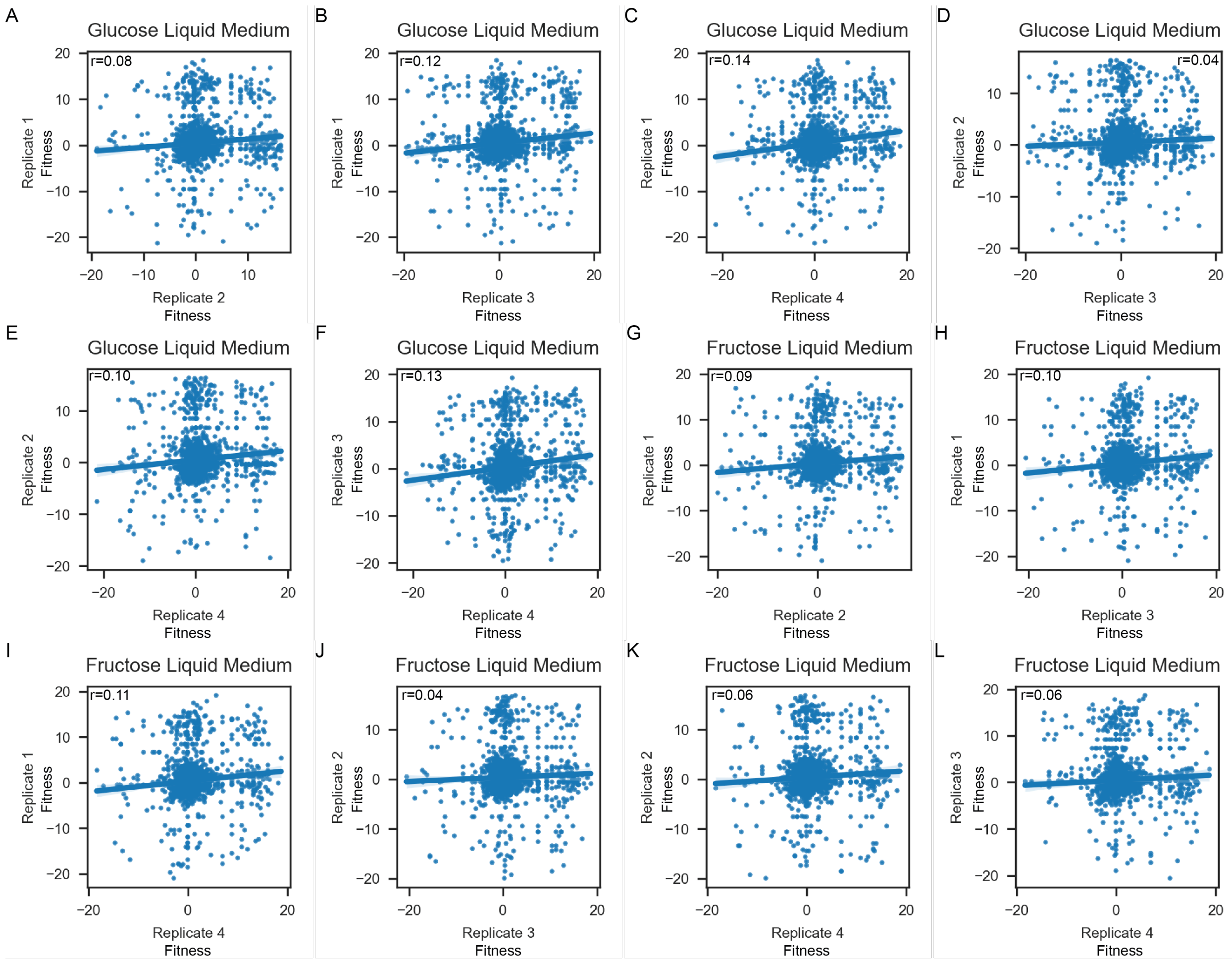

Fig S13. Comparison of BarSeq replicate gene fitness scores relative to T0 from the barcoded insertional mutant library grown on liquid minimal medium containing glucose or fructose as the sole carbon source. Comparison of fitness scores relative to T0 obtained from individual replicates of the insertional mutant library grown on (**A-F**) liquid minimal medium with glucose or (**G-L**) liquid minimal medium with fructose for 48 h; germlings were subsequently harvested for BarSeq. Genes were included in the fitness analysis if there were at least 10 reads associated with insertions in the gene. Lines are linear regression fits, and bands are 95% confidence intervals. R values for the linear regression lines are indicated on each graph ($p < 0.02$).

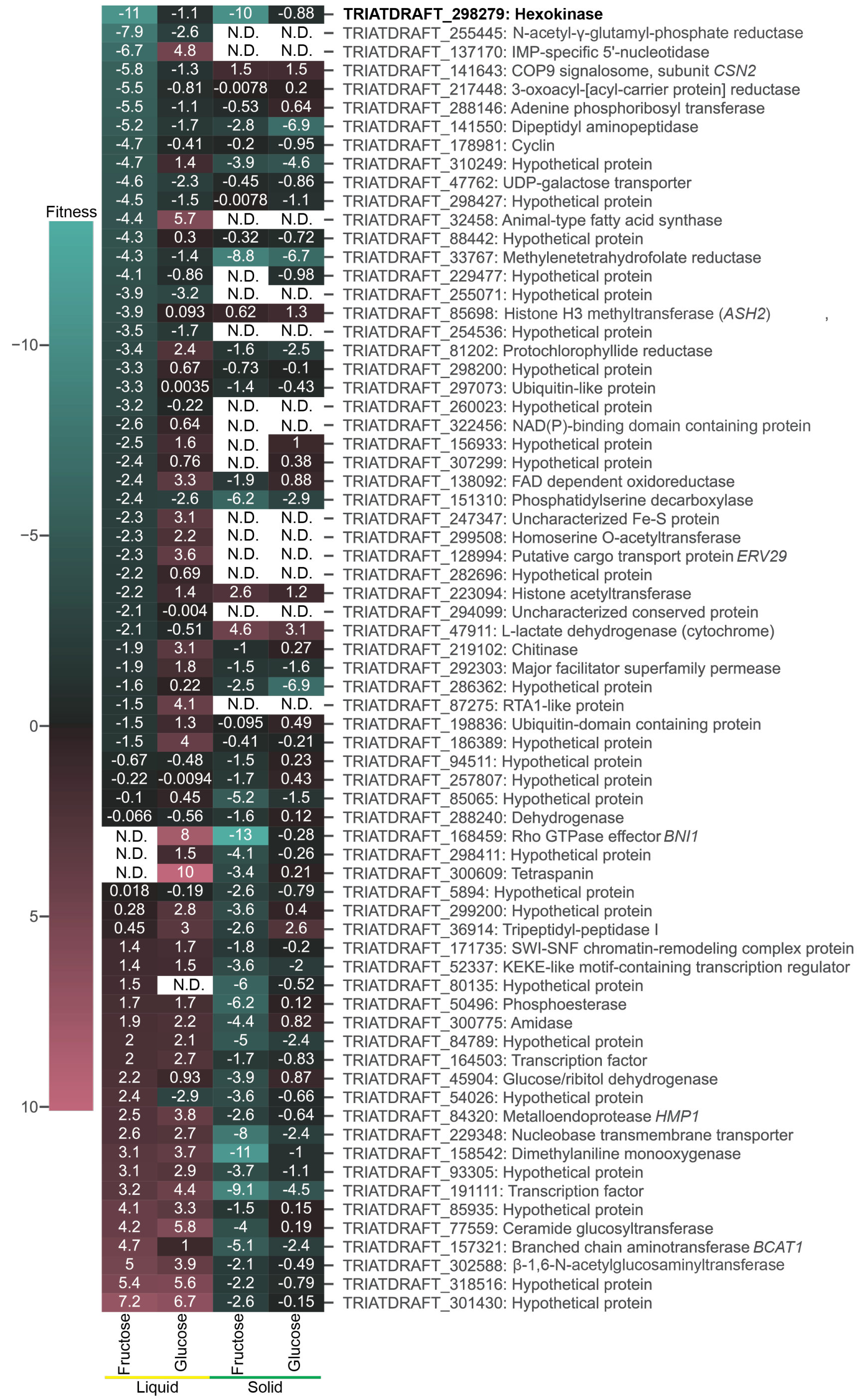

Fig S14. Identification of fructose utilization genes using BarSeq. Heatmap of fructose relative to T0 fitness scores of genes with fructose relative to T0 and fructose relative to glucose fitness scores below -1.5 with *t*-statistics < -3 in the liquid or solid fitness assay. A gene predicted to play a role in fructose utilization is indicated in bold. Fructose and glucose relative to T0 fitness scores are indicated on the heatmap; N.D. = No data, indicating that no barcodes associated with that gene had at least three counts across the reference condition and test condition or that the total number of barcode counts across all insertions in the gene was below 10 barcode counts in the reference and test condition.

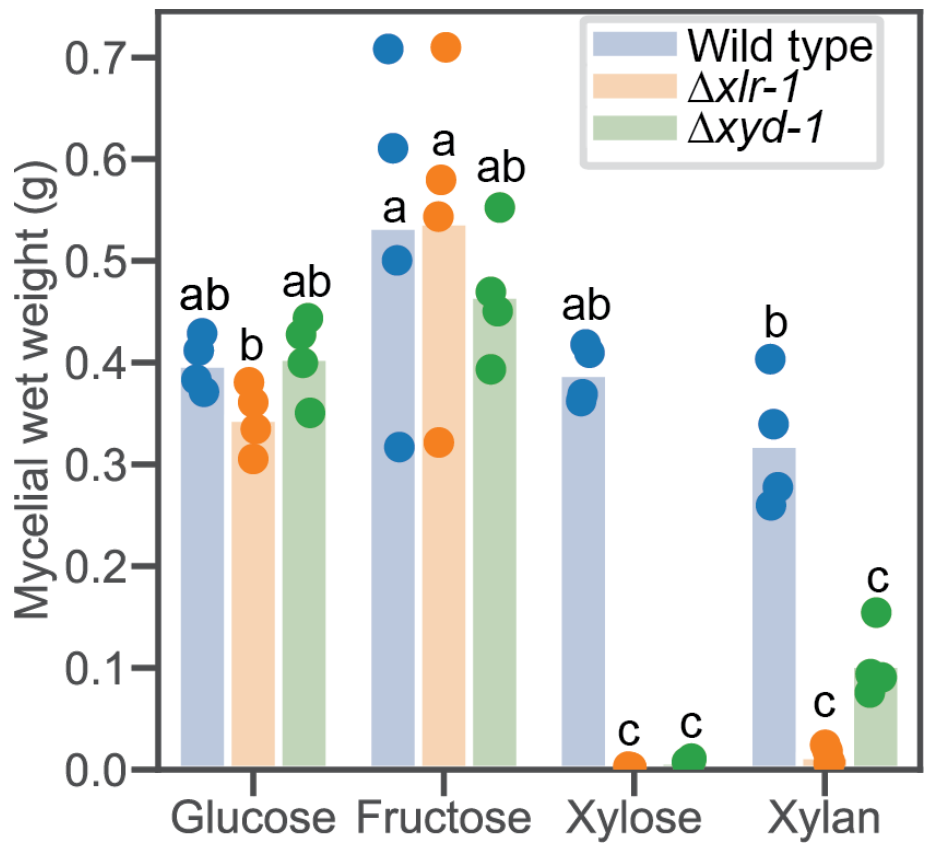

Fig S15. Phenotype of *Neurospora crassa* deletion mutants of orthologs of *T. atroviride* genes that showed fitness defects on xylose in *T. atroviride* BarSeq experiments. Mycelial wet weight of the indicated *N. crassa* deletion mutants (2, 3) of genes orthologous to the *T. atroviride* genes with xylose fitness defects that were also predicted to play a role in xylose utilization grown on the indicated carbon sources. Xylan is a hemicellulose polysaccharide composed primarily of β-1,4-linked D-xylose. Statistical significance was determined using one-way ANOVA, followed by Tukey's HSD test for pairwise comparisons ($p < 0.05$).

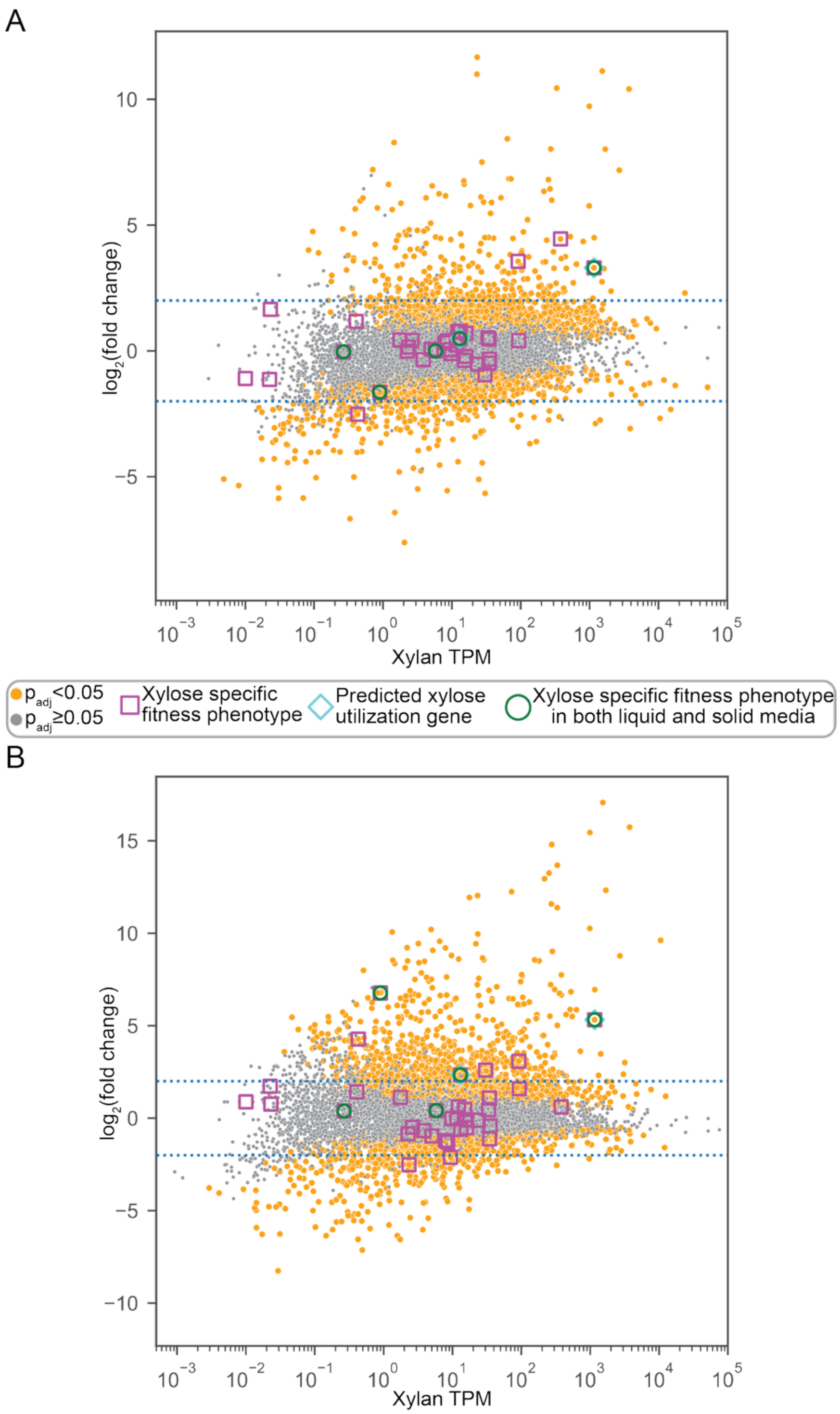

Fig S16. Differential expression analysis of *T. atroviride* exposed to xylan compared to glucose or carbon starvation. (A) Differential expression analysis of *T. atroviride* exposed to xylan versus carbon starvation. The x-axis indicates the transcripts per million (TPM) of *T. atroviride* genes during exposure to xylan. The y-axis indicates the $\log_2$(fold change) in gene expression during exposure to xylan as compared to carbon starvation. **(B)** Differential

expression analysis of *T. atroviride* exposed to xylan versus glucose. The x-axis indicates the transcripts per million (TPM) of *T. atroviride* genes during exposure to xylan. The y-axis indicates the log_2(fold change) in gene expression during exposure to xylan as compared to glucose. Genes with statistically significant differential expression ($p_{adj} < 0.05$) are indicated in orange. Genes without statistically significant differential expression ($p_{adj} \geq 0.05$) are indicated in grey. Genes with xylose-specific fitness phenotypes (i.e., xylose relative to T0 and xylose relative to glucose fitness scores below -1.5 with a *t*-statistic < -3 in either the liquid or solid fitness assay) are outlined with magenta squares. Genes with xylose-specific fitness phenotypes predicted to play a role in xylose utilization via homology are outlined with blue diamonds. Genes with xylose-specific fitness phenotypes in both liquid and solid media BarSeq experiments are outlined with green circles. Dotted blue lines indicate a log_2(fold change) in expression between cells exposed to xylan compared to (**A**) no carbon or (**B**) glucose of greater than 2 or less than -2.

SI Datasets

Dataset S1. The number of barcodes sequenced in the *E. coli* or *A. tumefaciens* plasmid libraries indicated as the number of barcodes with a given number of sequencing reads. The Chao estimate of the true library size (22) is estimated by the relative proportion of barcodes with 1 or 2 counts as described in the script Multicodes.pl (10).

Dataset S2. Barcode sequences in the barcoded *T. atroviride* insertional mutant library, their insertion location in the *T. atroviride* genome, and the total number of reads for each barcode. Types of barcoded insertions include: single (insertions that mapped exclusively to a single location in the *T. atroviride* genome), concat (i.e., concatemers: insertions that mapped to a single location in the *T. atroviride* genome and to the TDNA insertion sequence due to concatemers of TDNA insertion sequences in the same direction), or incat (i.e., inverted concatemers: insertions that mapped to a single location in the *T. atroviride* genome and to the TDNA insertion sequence due to concatemers of TDNA insertion sequences in opposite directions).

Dataset S3. *T. atroviride* barcoded insertional mutant library overview information: number of insertions in each scaffold (16), number of insertions in each genomic feature, and number of insertions per gene.

Dataset S4. Fitness scores, *t*-statistics, p-values, and the number of strains used to compute fitness scores for 2,797 genes obtained by growing the barcoded insertional mutant library on solid VMM (minimal medium) or solid minimal medium supplemented with all amino acids, adenine, uracil, p-aminobenzoic acid, and inositol (complete medium; VMM+) for 84 h and 2,772 genes obtained by growing the barcoded insertional mutant library on solid minimal medium supplemented with all amino acids, adenine, uracil, p-aminobenzoic acid, and inositol (complete medium) or solid minimal medium supplemented with adenine, uracil, p-aminobenzoic acid, inositol, and all amino acids except cysteine (complete medium without cysteine) for 84 h; conidia were subsequently harvested for BarSeq. Genes with a minimal medium-specific fitness phenotype had minimal medium relative to T0 and minimal medium relative to complete medium fitness scores < -1.5 with *t*-statistics < -3. Genes with a complete medium without cysteine-specific fitness phenotype had complete medium without cysteine relative to T0 and complete medium without cysteine relative to complete medium fitness scores < -1.5 with *t*-statistics < -3. Fitness scores of individual replicates are also included.

Dataset S5. Fitness scores, *t*-statistics, p-values, and the number of strains used to compute fitness scores for 3,321 genes that had a minimum of ten reads across all barcodes in the gene obtained by growing the barcoded insertional mutant library in liquid VMM with glucose or fructose as the sole carbon source for 48 h and 3,106 genes that had a minimum of 20 reads across all barcodes in the gene obtained by growing the barcoded insertional mutant library in liquid VMM with either glucose or xylose as the sole carbon source for 48 h; germlings were subsequently harvested for BarSeq. Fitness scores, *t*-statistics, p-values, and the number of strains used to compute fitness scores for 3,117 genes that had a minimum of ten reads across all barcodes in the gene obtained by growing the barcoded insertional mutant library on solid VMM media containing glucose or fructose as the sole carbon source and incubated for 84 h and 2,805 genes that had a minimum of 20 reads across all barcodes in the gene obtained by growing the barcoded insertional mutant library on solid VMM media containing glucose or xylose as the sole carbon source and incubated for 84 h; conidia were subsequently harvested for BarSeq. Genes with a fructose-specific fitness phenotype had fructose relative to T0 and fructose relative to glucose fitness scores < -1.5 with *t*-statistics < -3. Genes with a xylose-

specific fitness phenotype had xylose relative to T0 and xylose relative to glucose fitness scores < -1.5 with *t*-statistics < -3. Fitness scores of individual replicates are also included.

Dataset S6. Mycelial wet weight of the indicated *N. crassa* deletion mutants (2, 3) of genes orthologous to the *T. atroviride* genes with xylose fitness defects that are predicted to play a role in xylose utilization grown on the indicated carbon sources. Xylan is a hemicellulose polysaccharide composed primarily of β-1,4-linked D-xylose.

Dataset S7. Expression (transcripts per million; TPM) and differential expression calculated by DESeq2 (21) of the indicated comparisons of all *T. atroviride* genes during exposure to xylan, glucose, or media lacking a carbon source for 12 h. Gene fitness and xylose-specific phenotypes are calculated as described for Dataset S5.

Dataset S8. Primers used in this study.

SI REFERENCES

1. K. McCluskey, A. Wiest, M. Plamann, The Fungal Genetics Stock Center: a repository for 50 years of fungal genetics research. *J Biosci* **35**, 119-126 (2010).
2. H. V. Colot *et al.*, A high-throughput gene knockout procedure for *Neurospora* reveals functions for multiple transcription factors. *Proc Natl Acad Sci U S A* **103**, 10352-10357 (2006).
3. J. C. Dunlap *et al.*, Enabling a community to dissect an organism: overview of the *Neurospora* functional genomics project. *Adv Genet* **57**, 49-96 (2007).
4. H. Vogel, A convenient growth medium for *Neurospora* (medium N). *Microbial Genetics Bulletin* **13**, 42-43 (1956).
5. C. Engler, S. Marillonnet, Golden Gate cloning. *Methods Mol Biol* **1116**, 119-131 (2014).
6. S. T. Coradetti *et al.*, Functional genomics of lipid metabolism in the oleaginous yeast *Rhodosporidium toruloides*. *eLife* **7**, e32110 (2018).
7. E. P. Abbott, G. Ianiri, R. Castoria, A. Idnurm, Overcoming recalcitrant transformation and gene manipulation in Pucciniomycotina yeasts. *Appl Microbiol Biotechnol* **97**, 283-295 (2013).
8. M. Costello *et al.*, Characterization and remediation of sample index swaps by non-redundant dual indexing on massively parallel sequencing platforms. *Bmc Genomics* **19**, 332 (2018).
9. M. N. Price *et al.*, Oxidative pathways of deoxyribose and deoxyribonate catabolism. *mSystems* **4** (2019).
10. K. M. Wetmore *et al.*, Rapid quantification of mutant fitness in diverse bacteria by sequencing randomly bar-coded transposons. *mBio* **6**, e00306-00315 (2015).
11. M. Mersereau, G. J. Pazour, A. Das, Efficient transformation of *Agrobacterium tumefaciens* by electroporation. *Gene* **90**, 149-151 (1990).
12. P. Bundock, A. den Dulk-Ras, A. Beijersbergen, P. J. Hooykaas, Trans-kingdom T-DNA transfer from *Agrobacterium tumefaciens* to *Saccharomyces cerevisiae*. *EMBO J* **14**, 3206-3214 (1995).
13. M. J. de Groot, P. Bundock, P. J. Hooykaas, A. G. Beijersbergen, *Agrobacterium tumefaciens*-mediated transformation of filamentous fungi. *Nat Biotechnol* **16**, 839-842 (1998).
14. A. A. Wise, Z. Liu, A. N. Binns, Culture and maintenance of *Agrobacterium* strains. *Methods Mol Biol* **343**, 3-13 (2006).

15. J. J. Zik *et al.*, Dual transposon sequencing profiles the genetic interaction landscape in bacteria. *Science* **389**, eadt7685 (2025).
16. C. P. Kubicek *et al.*, Comparative genome sequence analysis underscores mycoparasitism as the ancestral life style of *Trichoderma*. *Genome Biol* **12**, R40 (2011).
17. J. Kim *et al.*, Multi-omics driven metabolic network reconstruction and analysis of lignocellulosic carbon utilization in *Rhodosporidium toruloides*. *Front Bioeng Biotechnol* **8**, 612832 (2020).
18. B. Bushnell, J. Rood, E. Singer, BBMerge - Accurate paired shotgun read merging via overlap. *PLoS One* **12**, e0185056 (2017).
19. A. M. Bolger, M. Lohse, B. Usadel, Trimmomatic: a flexible trimmer for Illumina sequence data. *Bioinformatics* **30**, 2114-2120 (2014).
20. R. Patro, G. Duggal, M. I. Love, R. A. Irizarry, C. Kingsford, Salmon provides fast and bias-aware quantification of transcript expression. *Nat Methods* **14**, 417-419 (2017).
21. M. I. Love, W. Huber, S. Anders, Moderated estimation of fold change and dispersion for RNA-seq data with DESeq2. *Genome Biol* **15**, 550 (2014).
22. A. Chao, Nonparametric estimation of the number of classes in a population. *Scandinavian Journal of statistics*, 265-270 (1984).
